# Hospitalisation trends of respiratory syncytial virus (RSV) infection in adults, six European countries, before and during COVID-19, 2016 to 2023

**DOI:** 10.2807/1560-7917.ES.2025.30.25.2400624

**Published:** 2025-06-26

**Authors:** Arantxa Urchueguía-Fornes, Richard Osei-Yeboah, Ombeline Jollivet, Caroline Klint Johannesen, Toni Lehtonen, Michiel van Boven, David Gideonse, Rachel A Cohen, Alejandro Orrico-Sánchez, Rolf Kramer, Thea K Fischer, Terho Heikkinen, Harish Nair, Harry Campbell, on behalf of the PROMISE investigators

**Affiliations:** 1 Vaccine Research Department, Foundation for the Promotion of Health and Biomedical Research in the Valencian Region (FISABIO), Valencia, Spain; 2 CIBER de Epidemiología y Salud Pública, Instituto de Salud Carlos III, Madrid, Spain; 3 Centre for Global Health, Usher Institute, The University of Edinburgh, Edinburgh, United Kingdom; 4 Sanofi, Lyon, France; 5 Department of Virology and Microbiological Preparedness, Statens Serum Institut, Copenhagen, Denmark; 6 Department of Clinical Research, North Zealand University Hospital, Capital Region, Copenhagen, Denmark; 7 Department of Health Security, Finnish Institute for Health and Welfare, Helsinki, Finland; 8 Centre for Infectious Disease Control, National Institute for Public Health and the Environment, Bilthoven, the Netherlands; 9 Julius Center for Health Sciences and Primary Care, University Medical Center Utrecht, Utrecht University, Utrecht, the Netherlands; 10 GSK, Rockville, United States; 11 Catholic University of Valencia, Valencia, Spain; 12 Department of Pediatrics, University of Turku and Turku University Hospital, Turku, Finland; 13 The members of the PROMISE investigators are listed under Acknowledgements

**Keywords:** Respiratory syncytial virus, adults, hospitalisation, surveillance, mortality, ICU admissions

## Abstract

**BACKGROUND:**

Respiratory syncytial virus (RSV) is a major cause of morbidity in older adults.

**AIM:**

We aimed to investigate the epidemiology of RSV in adults in five European countries and one region before and during the COVID-19 era.

**METHODS:**

We conducted a retrospective analysis using national hospital admission data from Denmark, England, Finland, the Netherlands, Scotland and regional prospective surveillance data from the Spain-Valencia region. We included patients aged ≥ 18 years hospitalised for respiratory tract infections (RTIs) 2016–2023 and assessed RSV-coded and laboratory-confirmed hospitalisations, intensive care unit (ICU) admissions and mortality.

**RESULTS:**

Hospitalisations associated with RSV varied by country and year but increased with increasing age regardless of the use of RSV-coded or RSV-confirmed data, the country or year. The highest hospitalisation rates were in patients aged ≥ 85 years. We found that RSV-coded hospitalisations underestimated the case numbers when compared with laboratory-confirmed cases by an average of 1.9 (standard deviation (SD): ± 0.9). Admissions to ICU associated with RSV in England and CFR in England and Finland displayed different patterns post-COVID-19 pandemic peak but were not notably higher compared with RTI admissions.

**CONCLUSION:**

Our findings reveal a consistency of RSV hospital admission patterns between European countries in the study period, with higher incidence rates among older patients. The differences between the numbers of RSV-coded and laboratory-confirmed cases highlight the critical need for improved surveillance, diagnostic practices and coding guidelines to better assess the incidence. Our findings could be vital for guiding public health strategies, particularly with the introduction of RSV vaccines for older adults.

**Key public health message**

**What did you want to address in this study and why?**

Respiratory syncytial virus (RSV) causes respiratory infections that can lead to hospital admissions and deaths. However, there is still a lack of understanding of how this disease affects adults. In this study, we aim to address this gap by evaluating hospital admissions resulting from RSV infections in adults in six European countries before and during the COVID-19 pandemic using routinely collected health data.

**What have we learnt from this study?**

We observed that RSV admissions increased with age, with the highest hospitalisation rates in adults aged ≥ 85 years in all countries. Data on cases without laboratory confirmation underestimated the number of patients hospitalised. In 2020/21 while there were strict measures in place to mitigate the COVID-19 pandemic, hospital admissions due to RSV notably reduced. However, in the subsequent years up to end of 2023, RSV admissions have started increasing again.

**What are the implications of your findings for public health?**

Our findings demonstrate that severe infections due to RSV in older adults display similar behaviours in the European countries included in the study. Improved surveillance and diagnostic practices are needed to better capture the true number of hospitalised patients infected with RSV. These findings are critical for guiding public health strategies, especially as RSV vaccinations for older adults are being implemented.

## Introduction

The impact of respiratory syncytial virus (RSV) in older adults is less understood than in young children. Recent findings show higher hospitalisation rates than previously acknowledged, particularly among adults with underlying health conditions, weakened immune systems or of advanced age [1-8]. The virus can exacerbate conditions such as asthma, chronic obstructive pulmonary disease (COPD) or heart failure, leading to severe outcomes, like pneumonia, myocardial infarction or death [9,10]. Yet, accurately determining the true incidence of RSV-related hospitalisations in older adults is challenging due to a lack of routine RSV testing and dedicated surveillance systems that include precise clinical criteria for the detection of patients with RSV [11-15].

The European Medicines Agency (EMA) has approved three RSV vaccines: in April 2023 Arexvy (GSK, London, the United Kingdom (UK)) for adults aged ≥ 60 years [16], in June 2023 Abrysvo (Pfizer, New York, the United States (US)) licensed for active immunisation of pregnant people and adults ≥ 60 years [17], and in August 2024, additionally approved an mRNA vaccine, mRESVIA (Moderna, Cambridge, US) [18]. With the wide range of RSV vaccines available for adults aged ≥ 60 years in Europe and the ones forthcoming [19], obtaining accurate RSV healthcare burden estimates in this population has become a public health priority to support immunisation strategies and monitor the impact and effectiveness of the new vaccines. The coronavirus disease 2019 (COVID-19) pandemic and associated non-pharmaceutical interventions (NPIs) had significant short-term impacts in lowering some respiratory viral circulations [20,21]. This reduction is known to have had an impact on RSV epidemiology including healthcare burden [22,23]. In 2021 and 2022, RSV returned after the strongest COVID-19 measures were lifted, but the patterns of returned RSV varied across geographies (e.g. out-of-season peaks, year-round hospitalisations).

We estimated hospitalisation rates, intensive care unit (ICU) admissions and mortality data of patients with RSV infections in five European countries (Denmark, England, Finland, the Netherlands, Scotland) and one region (Valencia region in Spain), as part of the PROMISE (Preparing for RSV Immunisation and Surveillance in Europe; https://usher.ed.ac.uk/respiratory-syncytial-virus-research/promise) initiative 2016–2023 [24].

## Methods

### Study design and population

A retrospective study of all-cause respiratory tract infection (RTI) admissions, RSV-coded admissions and RSV laboratory-confirmed admissions using routinely collected national hospital registries containing individual-level patient admission data from five countries (Denmark, England, Finland, the Netherlands and Scotland) and a regional prospective hospital-based active surveillance network in Spain-Valencia region [25,26]. Hospital admissions of patients aged ≥ 18 years with any mention of RTI using the International Classification of Diseases (ICD), ninth revision, clinical modification (ICD-9-CM) or ICD-10 codes (https://www.who.int/standards/classifications/classification-of-diseases) were extracted in Denmark, England, Finland, the Netherlands and Scotland. A full list of the codes is available in Supplementary Material. In Spain-Valencia, all hospital admissions for patients aged ≥ 18 years, arriving through the emergency room, meeting preliminary inclusion criteria (i.e. residents of the catchment area, not institutionalised and not hospitalised in the previous 30 days) and complying with the European Union influenza-like illness (ILI) [27] case definition were included. Demographic data in each country were obtained from national population information databases. Country-specific details are available in Supplementary Methods.

### Study period

The study period covered epidemiological years from 2016/17 to 2022/23; country-specific available study periods are described in Supplementary Methods. An epidemiological year was defined starting from the International Organization for Standardization (ISO) week 27 of a given year to ISO week 26 of the following year. In Spain-Valencia, the duration of the active monitoring period varied across years. To normalise for this variation and allow comparisons between epidemiological years, the study period was adjusted to the RSV circulation period as explained in Supplementary Methods.

### Outcomes

Hospital admissions were included if lasting > 8 hours in Spain-Valencia, > 12 hours in Denmark, Finland, the Netherlands and Scotland, and any duration for England. Day care, scheduled or routine admissions, as well as re-admissions within 28 days (30 days in Spain-Valencia) for the same diagnosis classification group, were excluded. The following hospital admission types were defined: (i) RTI-coded admissions, identified by any ICD-9-CM or ICD-10 code related to an RTI; (ii) RSV-coded admissions defined as any RTI admission including an RSV-associated ICD-9-CM or ICD-10 code (4801, 46611, 0796, J12.1, J20.5, J21.0, B97.4); (iii) RSV-confirmed admissions defined as any RTI admission, where a positive RSV polymerase chain reaction (PCR) test was performed within 7 days before to 2 days after the hospitalisation (data available only in Denmark, Finland, Scotland and Spain-Valencia). The number of RTI admissions for which an RSV-diagnostic test was performed was only known in Spain-Valencia.

A hospital admission was considered completed upon hospital discharge or death. In England, data on ICU admissions were also available. Mortality data included any death during hospitalisation (England) or during or after hospitalisation (Finland) where the primary or secondary causes of death included one of the diagnoses described in Supplementary Methods. For deaths occurring post-discharge, those that occurred > 14 days after the end of the hospitalisation were excluded. Hospitalisation types were further classified by the following diagnosis groups: upper respiratory tract infection (URTI), lower respiratory tract infection (LRTI) stratified by bronchitis and bronchiolitis, pneumonia and influenza, severe acute respiratory syndrome coronavirus 2 (SARS-CoV-2) or unspecified LRTI. More details can be seen in Supplementary Methods. Among RSV-related deaths, the primary cause of death was not available.

### Data analysis

Data from patients aged ≥ 18 years were analysed and stratified by the following age groups: 18–49 years (only in Finland and Scotland; in Finland denominators were not available for this age group), 50–64 years (only in Finland, Scotland and Spain-Valencia; in Finland no population data available for this age group), 18-64, 65–74, 75–84 and ≥ 85 years. Age was calculated as the person’s age at the time of each hospitalisation. Due to data privacy compliance, Denmark, Finland, the Netherlands and Scotland provided approximate counts when numbers were between 1 and 4, setting them to 2. Admission numbers were calculated by hospitalisation type, age group, diagnosis group, country and epidemiological year, and further stratified by the COVID-19 period. For the pre-COVID-19 and COVID-19 stratification, we excluded admissions from epidemiological years 2019/20–2020/21. The epidemiological year of 2020/21 was not considered representative, as NPI were at their strictest across all countries. This epidemiological year was not included in comparisons. In the epidemiological year of 2019/20, a notable number of RTI admissions were coded as SARS-CoV-2 across all regions (data not shown). Since the data were aggregated by full years — preventing the distinction between weeks with and without circulation — 2019/20 was excluded from the pre-COVID-19 group in all countries. We defined pre-COVID-19 admissions as the average (± standard deviation (SD)) of all admissions between 2016/17–2018/19 (from 2017/18–2018/19 for England and Scotland) and COVID-19 as the average (± SD) of all admissions between 2021/22–2022/23 (only 2021/22 for Denmark). The Netherlands was not included in the COVID-19 period due to data availability (until 2020/21). A sensitivity analysis including 2019/20 in the pre-COVID-19 stratification is included in Supplementary Material.

Admission to ICU and in-hospital case fatality ratios (CFR) were calculated using the corresponding hospitalisation numbers as denominators.

Admission rates and 95% confidence intervals (CIs) per 100,000 person-years for RSV-coded and RSV-confirmed hospitalisations were calculated by age group, country and epidemiological year using population data as of 1 January (Denmark, Finland, the Netherlands and Spain-Valencia) or mid-year (England and Scotland). The 95% CIs were estimated using a Poisson exact method. Person-years were calculated by multiplying the population by the duration of a full year, which was 1 for all countries except in Spain-Valencia where the population was multiplied by the duration of the RSV circulation period in years to account for annual differences in the duration of the active monitoring periods as shown in Supplementary Table 2. In Scotland and England, no population data for 2022/23 were available, so the population was assumed to be as the 2021/22 population. An average (± SD) of the study population and hospital admission rates was calculated over the whole study period for each age group and country, and further stratified by COVID-19 period. To understand RSV-coding practices, we divided RSV-confirmed admission rates by the corresponding RSV-coded rates for each country, epidemiological year and age group when enough sample size was available (≥ 10 patients). To quantify the impact of COVID-19 on admission rates for both RSV-coded and RSV-confirmed hospitalisations across countries and age groups, we fitted a Poisson regression model to compare rates between pre- and pandemic periods. From this model, we extracted the risk ratio (RR) along with its CIs and p value to understand if changes in admission rates during COVID-19 were significant, as presented in Supplementary Methods. Additionally, we conducted a sensitivity analysis by including the 2019/20 epidemiological year in the pre-COVID-19 era. To assess the consistency of trends across countries, we calculated pairwise Spearman’s correlation values between all country pairs for both RSV-coded and RSV-confirmed admissions, considering all epidemiological years (except 2020/21) and age groups together. We also compared the RSV-coded and RSV-confirmed datasets within each country among those with both types of hospital admissions available.

Data collection was standardised using common data collection templates and definitions. Country-specific data challenges prevented the use of a general definition for some of the outcomes, as detailed in Supplementary Methods. All analyses were performed in R (version 4.3.1) (https://www.r-project.org) using common programming scripts to ensure comparability despite healthcare systems heterogeneity.

## Results

The proportion of RSV-coded admissions among individuals aged ≥ 18 years during the whole study period ranged from 0.2% (Denmark) to 1.5% (Finland) (Table 1). Among countries with available laboratory-confirmed data, RSV-confirmed proportions ranged from 0.6% (Denmark) to 5.1% (Spain-Valencia) (Table 1). Overall numbers and proportions stratified by COVID-19 periods are available in Supplementary Table S1. Numbers and proportions of admissions stratified by age group, country, diagnosis group and COVID-19 are available in Supplementary Table S2. We categorised all RSV-coded and RSV-confirmed admissions by diagnosis group based on the recorded ICD codes at discharge. With few exceptions, pneumonia was the most common diagnosis group across age groups and epidemiological years in all countries (35–100%), followed by bronchitis and bronchiolitis or unspecified LRTI, depending on the country and epidemiological year. The proportion of URTI diagnoses was generally higher in the Netherlands and England compared with other countries. In Denmark and Finland, URTI diagnoses were more common when hospital admissions were RSV-confirmed rather than RSV-coded. More information is available in Supplementary Figure S1.

**Table 1:** Average population size and number of hospital admissions among patients with respiratory syncytial virus and aged ≥ 18 years, Europe, 2016–2023

Country	Study period	Average population	SD	RTI (n)	RSV coded (n)	%	RSV confirmed (n)	%
Denmark	2016/17–2021/22	4,652,883	47,104	235,230	477	0.2	1,393	0.6
England	2017/18–2022/23	44,321,027	360,008	4,305,867	21,587	0.5	NA	
Finland	2016/17–2022/23	4,479,585	35,360	337,751	5,078	1.5	6,259	1.9
The Netherlands	2016/17–2020/21	13,951,809	182,643	475,740	4,633	1.0	NA	
Scotland	2017/18–2022/23	4,431,017	23,027	272,105	1,185	0.4	2,057	0.8
Spain-Valencia	2016/17–2022/23^a^	378,995	51,303	8,752	48	0.5	442	5.1

NA: not applicable; RSV: respiratory syncytial virus; RTI: respiratory tract infection; SD: standard deviation.

^a^ Except 2020/21.

The average size of the population (± SD) aged ≥ 18 years during the whole study period is shown for each country. The total number of RTI admissions and the total number (and proportion from the RTI admissions) of RSV-coded and RSV-confirmed admissions among individuals aged ≥ 18 years are shown per country.

### Admission rates by epidemiological year, country and age

**Figure 1:**
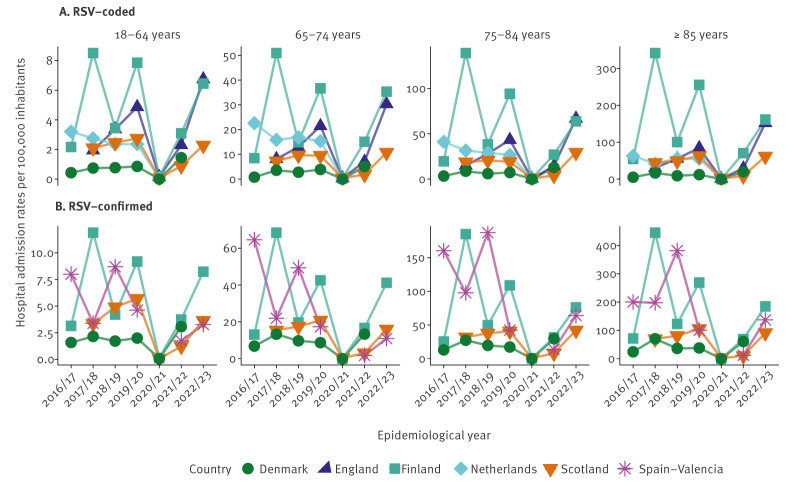
Rates of hospital admissions of patients with respiratory syncytial virus infection and aged ≥ 18 years, Europe, 2016–2023

Rates of hospitalisations of patients with RSV codes (per 100,000 person-years) varied by country, age and the COVID-19 period. Finland consistently had the highest RSV-coded admission rates across all ages (except in 2016/17), followed by England and the Netherlands (Figure 1A). All rates are available in Supplementary Table S5 and Supplementary Figure S2. In contrast, Denmark and Spain-Valencia reported the lowest rates. In Spain-Valencia, RSV-coded admissions were < 10 in each age group, Supplementary Table S2, so this region was excluded from the main analysis of RSV-coded admissions but included in Supplementary Material.

RSV: respiratory syncytial virus.

Epidemiological years availability: countries had varying data access availability, and not all epidemiological years of the study were fully available in all countries. In the Netherlands epidemiological years 2016/17, 2021/22 and 2022/23 were not available. In England and Scotland, 2016/17 was not available. In Denmark, 2022/23 was not available. In Spain-Valencia, 2020/21 was not available.

The hospital admissions rates per 100,000 persons-years (y-axis) and epidemiological year (x-axis) are shown for each country (colour), age group (vertical subpanels) and hospitalisation type. Note that each panel has a different y-axis scale to allow for readability. A summary figure with identical y-axis scales for all age groups can be found in Supplementary Figures S4 and S9. A tabular version of the figure displaying the numerical values (and 95% confidence intervals) is available as Supplementary Table S3 (RSV-coded incidence rates) and Supplementary Table S4 (RSV-confirmed incidences).

The lowest RSV-coded rates were consistently observed in individuals aged 18–64 years, with pre-COVID-19 average rates per 100,000 person-years ranging from 0.6 (SD: ± 0.2) in Denmark to 4.7 (SD: ± 3.4) in Finland, and rates during the COVID-19 period ranging from 1.4 (SD not computable) in Denmark to 4.8 (SD: ± 2.4) in Finland. Conversely, the highest RSV-coded rates were consistently observed among individuals aged ≥ 85 years, ranging from 10 (SD: ± 5.9) in Denmark to 166 (SD: ± 154) in Finland, per 100,000 person-years pre-COVID-19 and from 20 (SD not available) in Denmark to 116 (SD: ± 64.4) in Finland during COVID-19 (Figure 1A). More information is available in Supplementary Table S3, Supplementary Table S5, Supplementary Figures S2 and S4A. A table including average RSV-coded admissions in all countries and ages before and during COVID-19 is available as Supplementary Table S5. Annual and age-specific trends in RSV-coded admissions were highly correlated between all countries (Spearman correlation r^2^: 0.8–0.97), as presented in Supplementary Figure S5.

Among countries with information available on laboratory-confirmed RSV admissions, the admissions closely aligned with those observed in RSV-coded admissions (Spearman correlation r^2^: 0.96–0.99), but the observed hospitalisation rates were systematically higher in countries with access to laboratory data. Finland and Spain-Valencia displayed the highest RSV-confirmed admission rates in all age groups (Figure 1B). All rates are available in Supplementary Tables S3–S6 and Supplementary Figures S6–S7. In all countries, RSV-confirmed admission rates per 100,000 person-years increased with increasing age, with the lowest rates consistently observed among those aged 18–64 years (Figure 1B). In this age group, pre-COVID-19 average rates ranged from 1.8 (SD: ± 0.3) in Denmark to 7 (SD: ± 2.9) in Spain-Valencia, while average rates during COVID-19 ranged from 2 (SD: ± 1.7) in Scotland to 6 (SD: ± 3) in Finland. The highest rates were observed among individuals aged ≥ 85 years, varying from 44 (SD: ± 24) in Denmark to 260 (SD: ± 105) in Spain-Valencia pre-COVID-19, and from 51 (SD: ± 57) in Scotland to 127 (SD: ± 81) in Finland during COVID-19 (Figure 1B). A table including all countries and age groups average RSV-confirmed admissions before and during COVID-19 is available as Supplementary Table S6. Annual and age-specific trends in RSV-confirmed admissions were highly correlated between all countries (Spearman correlation r^2^: 0.6–0.94). More information is available in Supplementary Table S4, Supplementary Table S6 and Supplementary Figures S3 and S4B and S8.

A sharp decline in RSV admission rates was observed across all countries and age groups during 2020/21. During this period, the incidence of RSV-coded or RSV-confirmed admissions ranged between 0 and 1.6 per 100,000 person-years considering all countries and age groups (Figure 1). However, rates began to increase from 2021/22 onwards (Figure 1). More information is available in Supplementary Tables S3–S4 and Supplementary Figures S2–S4.

Risk ratios comparing RSV-coded and confirmed admission rates before and during COVID-19 differed. More information is available in Supplementary Tables S7–S10. In the RSV-coded admissions, a significant increase was observed only in England in individuals aged  ≥ 85 years (61%; p value < 0.001), whereas Finland saw a significant decrease of 48% (p value < 0.001) among those aged 75–84 years, and a decrease of 43% and 37% among individuals aged ≥ 85 years was observed in Finland and Scotland, respectively (p value < 0.05). More information is available in Supplementary Table S7. Changes observed in the rest of the countries or age groups during COVID-19 were not significant. In the case of RSV-confirmed admissions, a significant decrease during COVID-19 was only observed among patients aged ≥ 75 years in Finland (48%; p value < 0.05) and among those aged 65–74 and ≥ 85 years in Scotland (46% and 38%; p value < 0.05). In the case of Spain-Valencia, RSV-confirmed admissions showed a significant decline across all ages. Changes observed in the rest of countries or ages were not significant. More information is available in Supplementary Table S9.

In some countries (Finland, Spain-Valencia), high RSV admission rates in a given year were often followed by lower rates the year after (Figure 1). This pattern was also seen across some age groups in Denmark during pre-pandemic years. While hospital admission rate numbers varied between countries, a consistent increase with age was observed across all, regardless of the epidemiological year or COVID-19 stratification or whether RSV-coded or laboratory-confirmed data were used (Figure 1). More information is available in Supplementary Figures S2–S3, S9 and Supplementary Tables S3–S10.

### Coding practices

**Figure 2:**
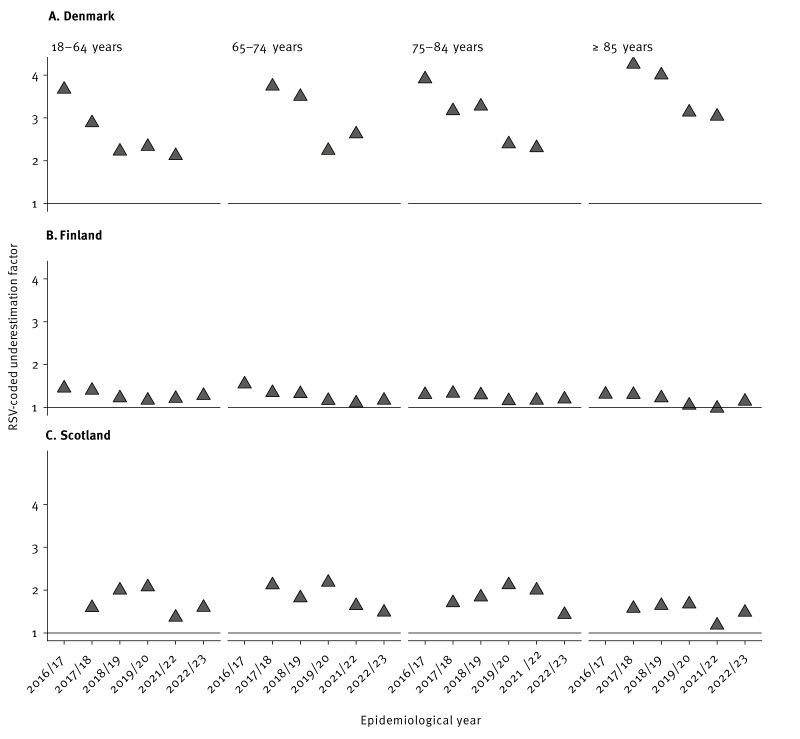
Underestimation of coding for respiratory syncytial virus infection in individuals aged ≥ 18 years, Denmark, Finland and Scotland, 2016–2023 (n = 16,417)

In countries with RSV-coded and RSV-confirmed data available, we estimated the degree of underestimation of RSV admission rates when relying solely on RSV codes. Denmark, Finland and Scotland had both types of data available and enough sample size (> 10 RSV-coded admissions). In these countries, regardless of the age group and epidemiological year, RSV-coded admissions resulted in an underestimation compared with RSV-confirmed admissions, except in 2021/22 in the ≥ 85 years age group in Finland (Figure 2). More information can be seen in Supplementary Figures S7 and S10. In Finland, the average underestimation factor when relying on ICD codes for RSV admission rate estimations was 1.3 (SD: ± 0.1) times pre-COVID-19 and 1.2 (SD: ± 0.1) times during COVID-19. In Scotland, the average underestimation factor was 1.8 (SD: ± 0.2) times pre-COVID-19 and 1.5 (SD: ± 0.2) times during COVID-19. Denmark showed the highest underestimation factor, with an average underestimation factor of 3.5 (SD: ± 0.6) times pre-COVID-19 and 2.5 (SD: ± 0.4) times during COVID-19. A figure including admissions of < 10 patients in the ICD-coded dataset is available as Supplementary Figure S10. Taking all countries and age groups together, on average, the use of ICD codes for the estimation of RSV admission rates underestimated the rate of admissions by 1.9 (SD: ± 0.9) times compared with RSV-confirmed admissions.

RSV: respiratory syncytial virus.

The RSV-coded underestimation factor (y-axis) has been obtained by dividing in each epidemiological year (x-axis), country (vertical subpanels) and age group (horizontal subpanels) of the corresponding RSV-confirmed admission rate by the corresponding RSV-coded admission rate. Numbers above the horizontal line (1) indicate that the incidence estimated with RSV-coded admissions is lower than the one estimated with RSV-confirmed admissions. Groups with < 10 patients in the RSV-coded dataset have been removed from this figure. A complete picture including groups with > 10 patients is available in Supplementary Figure S10.

### Admissions to intensive care units associated with respiratory syncytial virus infection in England

In England, data on ICU admissions were available and are summarised in Table 2. Across all ages, a higher ICU admission proportion among RSV-coded patients was observed pre-COVID-19 pandemic. Before COVID-19, among patients aged 18–64 and 65–74 years, the RSV-coded ICU admission proportions were higher than the overall RTI ICU-admission proportions (7.2% vs 5.8% and 7.9% vs 6.1%, respectively). This was not observed after 2020. The ICU admission proportion decreased with increasing age both among RTI-coded admissions and RSV-coded admissions. Numbers stratified by diagnosis group are available in Supplementary Table S9.

**Table 2:** Average number of admissions to intensive care units associated with respiratory tract infections (total n = 96,056) and infection with respiratory syncytial virus (total n = 458), by age, England, 2016/17–2018/19 and 2021/22–2022/23

Age (years)	Period	RTI-coded admissions (n = 96,056)			RSV-coded admissions (n = 458)		
		Average n	SD	%	Average n	SD	%
18–64	2016/17–2018/19	12,468	292	5.8	65	23	7.2
	2021/22–2022/23	10,318	5,387	3.8	47	7	3.0
65–74	2016/17–2018/19	7,372	332	6.1	46	7	7.9
	2021/22–2022/23	4,939	2,275	3.8	22	3	2.1
75–84	2016/17–2018/19	6,338	193	3.7	27	11	3.9
	2021/22–2022/23	3,716	1,380	1.9	12	4	0.9
≥ 85	2016/17–2018/19	1,996	97	1.2	5	0	0.9
	2021/22–2022/23	882	254	0.5	5	1	0.4

ICU: intensive care unit; RSV: respiratory syncytial virus; SD: standard deviation.

### Case fatality ratios associated with infection with respiratory syncytial virus in England and Finland

In countries with available mortality data, the CFRs among RSV-related hospitalisations (coded or laboratory-confirmed) varied by age group and country. The lowest RSV CFRs were observed in patients aged 18–64 years, ranging from 0.6% to 3.5%, while the highest RSV CFRs were observed in those aged ≥ 85 years, ranging from 6.2% to 17.6% (Table 3). England had higher RSV CFRs than Finland for both RTI-coded and RSV-coded admissions. In both countries, RTI-coded CFRs were consistently higher and across all ages compared with RSV-related (coded or confirmed) CFRs. Finland displayed the highest differences between the periods before and during COVID-19, with RSV-CFRs decreasing in all age groups 30–80% during the pandemic (Table 3). In England, a 6–47% increase in RSV-coded CFRs was observed during COVID-19. Such a decrease or increase among RTI-coded CFRs was not observed during COVID-19. Numbers stratified by diagnosis group are available in Supplementary Table S10.

**Table 3:** Average number of hospital admissions associated with respiratory tract infections (n = 363,577) and respiratory syncytial virus infection (n = 1,890) with a fatal outcome, by age, England and Finland, 2016/17–2018/19 and 2021/22–2022/23

Age (years)	Period	England						Finland								
		RTI deaths	SD	CFR (%)	RSV-coded deaths	SD	CFR (%)	RTI deaths	SD	CFR (%)	RSV-coded deaths	SD	CFR (%)	RSV-confirmed deaths	SD	CFR (%)
18–64	2016/17–2018/19	8,391	232	3.9	30	1	3.3	481	25	3.7	4	3	2.8	5	4	2.5
	2021/22–2022/23	10,550	1,368	3.9	54	42	3.5	402	227	3.5	1	1	0.6	2	0	1
65–74	2016/17–2018/19	12,660	612	10.4	43	3	7.4	995	28	8.6	7	8	4	8	10	3.5
	2021/22–2022/23	14,282	1,039	10.9	86	71	8.2	958	408	9.2	4	2	2	4	4	2.2
75–84	2016/17–2018/19	24,388	1,705	14.1	54	14	7.8	1,910	143	2.2	13	14	5.7	20	23	6.5
	2021/22–2022/23	28,196	26	14.6	152	148	10.8	1,736	687	11.5	7	7	3.5	8	8	3.4
≥ 85	2016/17–2018/19	34,054	3,645	19.8	69	20	11.9	2,559	163	17.8	30	27	12.3	38	42	12.3
	2021/22–2022/23	35,145	878	19.7	219	238	17.6	2,110	797	16.6	12	9	6.2	12	11	6.2

CFR: case fatality rate; RSV: respiratory syncytial virus; RTI: respiratory tract infection; SD: standard deviation.

The case fatality ratio to the total number of RTI or RSV-coded or confirmed admissions is also shown.

## Discussion

This study highlights the epidemiology of RSV in adults in six European countries, particularly in the context of the COVID-19 pandemic. The comparison between RSV-coded and RSV-confirmed hospitalisations revealed discrepancies in hospital admission rates, highlighting challenges in estimating healthcare burden of RSV-related illnesses. Despite these differences, both datasets exhibit consistent patterns of RSV circulation and age-specific hospitalisations between all included countries. This consistency suggests a broader applicability of the findings and provides valuable insights into RSV epidemiology despite the variability in healthcare systems, coding and testing practices. This is particularly relevant given the varying public health measures in place during the study period, including NPIs aimed at curbing COVID-19.

Hospital admission rates varied between countries and epidemiological years but consistently increased with increasing age regardless of the use of RSV-coded, RSV-confirmed data, the country or the epidemiological year. The highest rates were observed in patients aged ≥ 85 years with up to 445 laboratory-confirmed hospitalisations per 100,000 person-years. Biennial peaks of RSV admissions were noted in Finland, Spain-Valencia and Denmark, consistent with previous observations [28]. Similar findings have been seen in other regions worldwide, albeit most studies focus on high-income countries [29-31]. During 2020/21, when the strongest COVID-19 NPIs were in place, almost no RSV-associated hospitalisations occurred. Although country-specific nuances were observed in hospital admission rates, England, Finland, the Netherlands and Scotland displayed similar annual and age-specific RSV-coded admission trends (Spearman correlation r^2^: 0.79–0.97). Higher incidences were consistently observed among countries with access to laboratory data (Denmark, Finland, Scotland and Spain-Valencia). The highest RSV-confirmed admissions were observed in Spain-Valencia, where testing was systematic, and in Finland, likely reflecting broader testing practices, although testing denominators were only available in Spain-Valencia, making the assessment of testing practices difficult. It is important to note that despite the availability of PCR laboratory-confirmed data; RSV incidence may still be underestimated by at least 2.2 times due to the underestimation of diagnostic tests based on RT-PCR [32,33]. Similar to RSV-coded admissions, high correlations (Spearman correlation r^2^: 0.75–0.94) were observed across all countries with access to laboratory data, reflecting consistent age-specific admission rates. In routinely collected healthcare records, laboratory test results are often unavailable, and even when accessible, testing denominators are typically unknown. As a result, RSV-coded data serve as the best available proxy to be included in estimations of RSV hospitalisation numbers. Our analysis found that reliance on RSV codes led to underestimation factors ranging from 1.1 (Finland) to 4.3 (Denmark). Denmark had the lowest RSV-coded admission rates, consistent with previous research showing that ICD-coded admissions underestimate case numbers compared with RSV-positive hospitalisations in Denmark [26]. However, despite this underestimation, RSV-coded and RSV-confirmed admissions exhibited similar patterns in Denmark, Finland and Scotland, with r^2^ correlations ranging from 0.96 to 0.99. This suggests that while RSV-coded admissions underestimate the case numbers, they still reliably capture numbers of hospitalised patients and can serve as a useful epidemiological indicator when laboratory data are unavailable in adults.

Our analysis of admission rates before and during COVID-19 revealed different results between age groups, countries and data collection methods (RSV-coded/RSV-confirmed). For instance, in the RSV-coded dataset, there was a significant increase in admissions in individuals aged ≥ 85 years but not in adults of other ages in England, while in Finland and Scotland there was a significant decrease in admissions among some age groups (75–84 and ≥ 85 years). In the case of RSV-confirmed admissions, we observed a significant decrease during COVID-19 in the older age groups only in Finland and Scotland. In Spain-Valencia, a significant decrease was observed among all ages. Internal assessments revealed that there were no changes in the proportions of admitted and tested patients between the two COVID-19 eras in Spain-Valencia, meaning that testing rates and inclusion criteria were not altered (data not shown). Due to the nature of the data collected, fully understanding the source of the observed differences is currently challenging. The varying results could reflect low infection rates due to NPI changes, variations in testing or coding practices between countries, or changes in healthcare-seeking behaviours, among others. Initially, we excluded 2019/20 in the COVID-19 stratification. Including 2019/20 in the pre-COVID-19 era did not alter the overall observed changes during COVID-19 (increase/decrease) or the estimated average incidence rates pre-COVID-19; however, in a few age groups and countries, it did affect the significance of the changes. It is important to note that our data included up to four epidemiological years pre-COVID-19 and two during COVID-19, which included 2021/22, where NPI were still in place in most countries [34]. This may have impacted the estimates. Data on more epidemiological years post-COVID-19 is needed to fully understand trends after 2020.

Admissions to ICU associated with RSV were slightly higher among RSV-coded patients compared with general RTI admissions pre-COVID-19, but not after 2020, suggesting a potential impact of COVID-19 in increasing the severity of RTI admissions (where COVID-19 diagnoses might have been included). A decrease in ICU admissions was observed both among RTI and RSV-coded admissions after 2020, which could reflect limited ICU capacity. The observed CFRs decreasing in Finland and increasing in England are likely a reflection of the changes in coding or testing practices observed after 2020 as discussed previously. Since vaccination can significantly reduce hospitalisation, ICU admission rates and fatal outcomes, a direct comparison between all-cause RTI and RSV admissions without accounting for vaccination status, may not accurately reflect the true incidence of RSV. For instance, available vaccines for other respiratory pathogens, like influenza, have been shown to reduce the severity of illness in vaccinated individuals, leading to decreased hospitalisation and ICU admissions [35]. In contrast, the absence of a vaccine for RSV during the study period may contribute to a higher healthcare burden of severe cases, with studies indicating higher requirements of ICU care [36].

Among both RTI-coded and RSV admissions, CFRs increased with increasing age and ICU admissions decreased with increasing age. Older adults are more susceptible to severe outcomes following hospitalisation, which, in addition to the increased risk of death with age [37], may explain the observed CFR age-specific patterns. The decline in ICU admissions among older adults could be attributed to different causes, for example to age-based triage decisions prioritising younger patients when ICU capacity is limited or to the known decreased chances of ICU survival in older adults after ICU stays [38,39]. Understanding which scenario may apply to the observed ICU age-admission patterns in England requires further research.

Our study presents several limitations that must be considered when interpreting the findings. The data collection method in Spain-Valencia differed as it relied on an active surveillance network setup for influenza surveillance and on the ILI case definition, which is well-known to underestimate the real RSV healthcare burden [11,14,40]. Moreover, data in each year was adjusted to the RSV circulation period and the admission rates were shown per 100,000 adults during the RSV circulation period. It also represents a region in Spain with a population size similar to that of Scotland. However, the results should be interpreted at the regional level rather than extrapolated to the entire country. We did not use RSV-coded admissions in the main analysis as only three diagnoses were recorded at discharge. Nevertheless, because testing was systematic across all included patients, the addition of the RSV-coded dataset did not bring additional value. In this region, mortality data were also available, but the average number of RTI deaths in the dataset was too low for a correct interpretation of RSV-coded and RSV-confirmed deaths. The low number of severe outcomes may indicate challenges in reaching critically ill patients through active surveillance, making rare cases less reliable and unlikely to accurately reflect the region's reality. Despite these differences, the inclusion of data from this region allowed us to measure the impact of a systematic testing strategy in the incidence rate estimations and we found that despite the variability in the data collection method, similar trends in rates and age distribution of RSV-confirmed cases were observed between countries with RSV-confirmed data and Spain-Valencia (Spearman correlation r^2^: 0.6–0.93). This comparison further highlights the fact that routinely collected national healthcare data provide valuable insights into RSV trends and epidemiology in adults.

Nevertheless, the reliance on routinely collected national healthcare data introduces potential biases, including coding inaccuracies and inconsistencies in clinical practices across different countries. Particularly, the ICD-coded admissions likely underestimate the true healthcare burden of RSV, as evidenced by underestimation factors ranging from 1.1 times in Finland to 4.3 times in Denmark. On the other hand, Scotland had only six available ICD codes at discharge, which may have impacted the number of included patients with RSV. Our analysis included an uneven number of years for the periods before and during COVID-19 which in combination with the alternating patterns of high and low RSV admissions in several countries may have impacted the number of RSV-coded or RSV-confirmed cases. The disparity in coding practices, the incomplete understanding of RSV testing practices, and the uneven number of years displaying alternating patterns of low and high admissions complicates the analysis and makes the understanding of the changes post COVID-19 observed difficult to interpret and contextualise. The variability in primary data collection methods and the limited scope of laboratory-confirmed cases restrict direct comparability across countries. These limitations underscore the need for more systematic and standardised testing approaches and general coding guidelines to improve the accuracy and reliability of estimates of RSV case numbers at the European level. The use of other approaches for RSV related hospitalisation estimations such as time-series modelling [41] could help in overcoming some of these limitations. The lack of granular data hindered a more detailed analysis of admission rates among individuals with comorbidities, such as those with weakened immune systems, who are known to be at higher risk of severe RSV infections, as highlighted by a recent modelling study [42]. Hence, more specific data on individuals with comorbidities is still needed to fully understand the impact of RSV. Moreover, limiting the inclusion criteria to respiratory codes likely underestimates the true incidence, as respiratory infections can manifest with symptoms beyond respiratory [10,43]. The fact that among RSV-related deaths, the primary cause of death was not available, limits the interpretability of the CFRs. Finally, the lack of data stratification by sex is another limitation. A recent study that used the active surveillance network in Spain-Valencia to estimate RSV hospitalisation rates among adults found no differences by sex in RSV hospital admissions [15]. In many countries, severe acute respiratory illness (SARI) surveillance has been implemented [44] or recommended for RSV [45], and we hope this study will contribute to contextualising the results of SARI surveillance, enhancing the understanding of RSV epidemiology in the future.

## Conclusion

This study provides a comprehensive overview of RSV-associated hospitalisations in adults across five European countries and one region, offering critical insights into RSV epidemiology and severity in the context of the COVID-19 pandemic. The findings were consistent between countries and indicative of the public health burden posed by RSV in adults. Individuals aged ≥ 85 years consistently displayed the highest hospitalisation rates in all the countries and years studied. The differences observed between RSV-coded and laboratory-confirmed cases highlight the critical need for improved surveillance, diagnostic practices and coding guidelines. The introduction of new RSV immunisation strategies underscores the necessity for reliable epidemiological data to guide public health policies and interventions. As such, this study lays the groundwork for future research and surveillance efforts aimed at mitigating the impact of RSV, particularly in the context of evolving viral dynamics and public health landscapes.

## Data Availability

All data are presented in this manuscript. Written requests may be made to the relevant data controllers to access specific national or regional data.

## Supplementary Data

2378000 Supplement
