# Supplementary material for: Hospitalisation trends of respiratory syncytial virus (RSV) infection in adults, six European countries, before and during COVID-19, 2016 to 2023
Source: Euro Surveill. 2025 Jun 26;30(25):2400624. doi: 10.2807/1560-7917.ES.2025.30.25.2400624 (PMC12207200; doi:10.2807/1560-7917.ES.2025.30.25.2400624)
Supplement: Supplement [file 24-00624_OSEI-YEBOAH_Supplement.pdf]

Internal

Supplementary Materials

This supplementary material is hosted by Eurosurveillance as supporting information alongside the article [Hospitalisation trends of respiratory syncytial virus (RSV) infection in adults, six European countries, before and during COVID-19, 2016 to 2023], on behalf of the authors, who remain responsible for the accuracy and appropriateness of the content. The same standards for ethics, copyright, attributions and permissions as for the article apply. Supplements are not edited by Eurosurveillance and the journal is not responsible for the maintenance of any links or email addresses provided therein.

Methods

1. National registries

Denmark

The Danish data is based on the Danish National Patient Registry (NPR) [1-4]. The NPR provides nationwide longitudinal registration of detailed administrative and clinical data [1-4]. For each patient contact, one primary and numerous, optional, secondary diagnoses are recorded. For this study, data on all admissions with an RSV diagnosis were gathered from 2016 to 2022. Linkage to RSV-positive laboratory confirmations was possible through data from the national clinical microbiological database, where data from all microbiological tests are stored. This data was provided by Statens Serum Institute through the KIDS database. Patients identified in DNPR were also linked to the Medical Birth Registry (MBR), as well as the Civil Registration System (CRS) to obtain additional and missing information. The MBR contains data on all births in Denmark since 1973 including background information on the circumstances of the birth. No information on SARS-CoV-2 was available. When the number of admissions or deaths was between 1 and 4, information was provided as “<5” and treated in the analysis as 2 to enable display on one hand and aggregation over multiple values of the other hand. Demographic data for population estimates were collected from Statistics Denmark [5].

England

Hospitalisation and death data were collected from the England Hospital Episode Statistics (HES) [6] database that monitors >98% of England's population and gathers information related to the patient and their clinical course in the hospital. HES is a reimbursement dataset that facilitates secondary care provider reimbursement from local health commissioners. HES contains details of inpatient, including ICU, admissions in a dataset called Admitted Patient Care (APC), from National Health Service (NHS) hospitals in England. Patient secondary care activity is longitudinally linked across all NHS hospitals. The information within HES is collected across services as part of the Commissioning Data Sets. Data are submitted monthly to NHS Digital for processing and is made available as the Secondary Uses Service dataset which can be used for non-clinical purposes, such as research and planning health services. In HES, APC diagnoses are recorded using the ICD-10 and procedures performed using the Office of Population, Censuses and Surveys Classification of Surgical Operations and Procedures, fourth revision (OPCS-4). Inpatient admissions may have up to 20 ICD-10 diagnoses, of which one is the primary cause of admission, investigation, or treatment, and the remaining 19 codes are secondary diagnoses codes. The same is available for procedures with up to 20 OPCS-4 codes. ICU episodes can be level two (high dependency) and level three (intensive organ support and/or nursing care). ICU episodes can occur multiple times during the same overnight admission as patient care is escalated and de-escalated. When a patient dies during a hospital stay, this is recorded in the HES APC dataset as an inpatient death. However, deaths that occur among patients outside of the inpatient setting are not recorded in this dataset. To exclude scheduled admissions, the HES admission method field was used, and all hospitalisations that were labelled as "Waiting list", "Booked", or "Planned" were excluded. The exclusion criterion for admissions shorter than 12 hours was not applied, as the admission duration in HES can only be calculated in whole days. Day-care hospitalisations were excluded by filtering the HES Admitted Patient Care data table on inpatients. In addition, the inclusion criteria filtered unplanned inpatients. Admissions coded as

COVID-19 that occurred prior to 01 March 2020 were excluded, as these were likely to be coding errors. Where the age was missing, it was imputed using information from any other admissions for that person where the age was recorded. From this, an approximate date of birth was calculated and then used to calculate an approximate age at the time of the admission with the missing age. When nothing was available to impute age, the admission was counted in the “Overall” age group category only. The earliest occurrence of each risk factor was identified for each person, using all HES admissions, including non-RTI admissions. People were considered to have that risk factor from that point forward. Any admission occurring at or after the risk factor was first observed was counted under that risk factor. The population comes from various United Kingdom censuses from the National Office for Statistics: for 2021/2022 the population described in Census 2021 [7], for previous seasons’ populations the mid-year census of the year, i.e., week 27 of the season [8]. For 2022/2023, as the census was not yet publicly available at the time of the study, we assumed the same population for mid-2022 as the one in mid-2021 to compute the incidence, which is expected to have low bias on the outcomes as the population is not varying a lot over years.

Finland

The Finnish data is based primarily on two nationwide registers maintained by the Finnish Institute for Health and Welfare (THL): the Finnish Care Register for Health Care (HILMO) and the National Infectious Diseases Register (NIDR). HILMO covers individual level clinical and administrative data from inpatient care, specialised outpatient care and day surgeries from both hospitals and other institutions. Each registered event has a symptom-cause pair of primary diagnoses and similar pairings of optional secondary diagnoses, all of which have been recorded using the ICD-10 classification since 1996. For this study the data from HILMO was gathered from 2016 to 2023. The NIDR captures records of selected microbiological findings and related diagnoses that all laboratories and physicians are obliged to report under the Communicable Disease Act. All positive test results for RSV, influenza A virus and influenza B virus are included in the register, though there is no denominator information on the total number tested. General demographic records and dates of

death for the study population were obtained from the Population Information System which is maintained by Statistics Finland. The linkage of data from different registers was carried out using unique personal identification codes.

Scotland

In Scotland, we used the Scottish Morbidity Record (SMR01) and Electronic Communication of Surveillance in Scotland (ECOSS) registries containing individual-level patient data on all inpatient and day cases in hospitals and laboratory cases. We used the continuous inpatient stay (CIS) marker to group admissions belonging to the same episode; for example, transfers from one hospital or significant facility to another were included as the same admission. The period of data availability was between January 2017 to June 2023, and we only integrated cases within the defined surveillance seasons (week 27 of year Y to week 26 of year Y+1). The study was approved by the Public Benefit and Privacy Panel for Health and Social Care (HSC-PBPP), and data were provided by the electronic Data Research and Innovation Services (eDRIS) [9, 10]. Mid-year population estimates for Scotland was obtained from the National Records of Scotland [11].

Netherlands

The Dutch Hospital Data (DHD) registration collects, manages and processes hospital data and manages standards for its registration. The used data sources were LBZBASISTAB and LBZDIAGNOSENTAB for the years 2016 to 2021. All diagnoses were recorded using ICD-10 classification. The demographic database from Statistics Netherlands (CBS) GBAPERSOONTAB was used to link data on gender and birth date to the admitted patients. Linkage to RSV-positive confirmations was not possible for this study. This non-public microdata was provided by Statistics Netherlands [12].

Spain-Valencia

In Spain-Valencia, patient information was collected through the Valencia Hospital Surveillance Network for the Study of influenza and other Respiratory Viruses (VAHNSI), an active prospective

hospital-based surveillance network (~1 million catchment population; 21% of the total Valencia population). The network is coordinated by the Vaccine Research Department of FISABIO-Public Health and has been described previously [13-16]. Patients had to fulfil the following criteria to be included in the study: being hospitalised via emergency room with a diagnosis compatible with an RTI, residing in the catchment area of one of the participating hospitals for at least 6 months, non-institutionalised, not discharged from a previous hospital admission in the last 30 days and give their (or their legally authorised representative) written consent. Patients ≥ 18 years old were included if, upon admission, they met symptoms compatible with the Influenza-like-Illness case definition, defined as the presence of at least one respiratory symptom (cough, sore throat or shortness of breath) with an onset within 7 days prior to admission [17]. As VAHNSI is a surveillance network initially set up to cover influenza seasons, monitoring did not occur throughout the whole year and the duration of the monitoring was also different across seasons. Therefore, data was adjusted to the RSV circulation period in each of the season to allow data comparison across years with different surveillance lengths. Circulation was defined as the weeks between the first of at least two consecutive weeks with two or more RSV cases and the week before the first of at least two consecutive weeks without RSV cases considering the PCR results of included patients from all ages. The loss of RSV confirmed cases after adjusting the data to the circulation period seasons is negligible. The duration in weeks of each season was calculated as the total number of epidemiological weeks in each RSV circulation period. The population was adjusted to the length of the RSV circulation period in years (approximated as 1 year; ~ 52.143 weeks). Table 1 and 2 describe the hospitals involved and the RSV circulation period in each season. Due to the COVID-19 pandemic no data was collected during season 2020/2021. During 2021/22 there were two RSV circulation periods (from W43 to W05 and from W13 to W26) and data from both circulations have been used in the study. Demographic information was obtained from The Population Information System (Sistema de informacion Poblacional, SIP) [18], a region-wide database that provides basic information on sociodemographic and administrative data.

Internal

Table 1 Participating hospitals and their overall catchment size

Hospital	Season	Catchment size
General Castellón	All	279,111
La Fe	All	284,152
Dr. Peset	All	272,842
General Alicante	Until 2019/20	271,120
La Marina Baixa	2021/22 and 2022/23	184,076

Table 2 Data collection period per season in Spain-Valencia

Season	RSV-circulation starts	RSV-circulation ends	N weeks	Length (years)
2016/17	2016-W45	2017-W11	18	0.34
2017/18	2017-W44	2018-W17	25	0.48
2018/19	2018-W44	2019-W14	22	0.42
2019/20	2019-W44	2020-W11	19	0.36
2021/22**	Circ1: 2021-W43<br>Circ2: 2022-W13	Circ1: 2022-W05<br>Circ2: 2022-W26	28	0.54
2022/23	2022-42	2023-12	22	0.42

** Two RSV circulation periods

No follow-up of patients was done after hospital discharge.

The study protocol was approved by the Ethics Research Committee of the Dirección General de Salud Pública-Centro Superior de Investigación en Salud Pública (DGSP-CSISP). All subjects signed written informed consent prior to their inclusion in the study.

2. Country-specific data availability

Table 3 summarises the data availability of each of the participating countries

Table 3 Data availability per country.

Data availability	Scotland	England	Netherlands	Finland	Denmark	Spain-Valencia
**Population coverage**	National	National	National	National	National	~21% Spain-Valencia region
**Denominator population of ≥18-year-olds [Mean (range) over study period]**	4,431,017 (4,394,745, 4,454,919)	44,321,027 (43,752,473, 44,715,443)	13,951,809 (13,701,285, 14,190,874)	4,479,585 (4,431,392, 4,537,778)	4,652,883 (4,580,547, 4,721,691)	378,995 (306,076, 456,659)
**Years available for this study**	2017-2023	2017-2023	2016-2021	2016-2023	2016-2022	2016-2023 (except 2020/21)
**Linkage to RSV laboratory confirmations**	Yes	No	No	Yes	Yes	Yes
**Coding system**	ICD10	ICD10	ICD10	ICD10	ICD10	ICD-9, ICD10
**Maximum number of diagnosis codes per record**	6	20	No maximum	No maximum	No maximum	3
**Mortality data**	No	Yes	No	Yes	Yes	Yes
**ICU admissions**	No	Yes	No	No	No	No
**Risk group identification**	No	Yes	No	Yes	Yes	Yes

3. List of ICD codes

In Table 4, the list of ICD-10 and ICD-9 codes used to identify RTI and RSV-coded (in red) hospitalisations. The codes are shown together with their corresponding diagnosis group classification.

Table 4 List of ICD-10 and ICD-9 codes used in this study, with RTI codes in black and RSV codes in red.

Diagnosis classification	ICD-10	ICD-9
**Acute upper respiratory tract infection (URTI)**	J00 J02.0 J02.8 J02.9 J03.0 J03.8 J03.9 J04.0 J04.1 J04.2 J05.0 J05.1 J06.0 J06.8 J06.9	460 4610 4611 4612 4613 4618 4619 462 463 46400 46401 46410 46411 46420 46421 46430 46431 4644 46450 46451 4650 4658 4659
**Pneumonia & Influenza (LRTI)**	J09 J10.0 J10.1 J10.8 J11.0 J11.1 J11.8 J12.0 **J12.1** J12.2 J12.3 J12.8 J12.9 J13 J14 J15.0 J15.1 J15.2 J15.3 J15.4 J15.5 J15.6 J15.7 J15.8 J15.9 J16.0 J16.8 J17.0 J17.1 J17.2 J17.3 J17.8 J18.0 J18.1 J18.2 J18.8 J18.9	4800 **4801** 4802 4803 4808 4809 481 4820 4821 4822 48230 48231 48232 48239 48240 48241 48249 48281 48282 48283 48284 48289 4829 4830 4831 4838 4841 4843 4845 4846 4847 4848 485 486 4870 4871 4878 514
**Bronchiolitis & Bronchitis (LRTI)**	J20.0 J20.1 J20.2 J20.3 J20.4 **J20.5** J20.6 J20.7 J20.8 J20.9 **J21.0** J21.1 J21.8 J21.9 J40	4660, **46611**, 46619
**Unspecified LRTI**	J22	5198 (not exact map)
**SARS-CoV-2/COVID-19**	U07.1, U07.2, U08-10	-
**RSV-specific codes**	**B97.4**	**0796**

4. Statistical comparisons between the pre and post-COVID-19 periods

To quantify the impact of COVID-19 on admission rates for both RSV-coded and RSV-confirmed hospitalisations across countries and age groups, we fitted a Poisson regression model to compare rates between pre- and post-COVID-19 periods. We used the generalized model function (*glm*) from R with the following structure for each hospitalisation type:

$$\lambda_{i,j} \sim covid_{i,j} + offset\left(\log\left(P_{i,j}\right)\right)$$

where

λ is the expected value of hospitalisation counts for country i and age group j ($\lambda_{i,j}$ response variable).

$covid_{i,j}$ is the independent predictor variable for country i and age group j, 0 for pre and 1 for post-COVID-19.

P is the population, included as an offset using its logarithm for country i and age group j $(P_{i,j})$

The family is Poisson, meaning the response variable Y follows a Poisson distribution:

$Y_{i,j}$~ Poisson($P_{i,j}$ * $\lambda_{i,j}$), assuming the following Poisson model for the expected incidence rate ($\frac{\lambda_{i,j}}{P_{i,j}}$):

$$\log(\lambda_{i,j}) = \beta_0 + \beta_1 * covid_{i,j} + \log(P_{i,j})$$

where:

β_o, β_1 are the coefficients of the model associated with the covid variable.

The exponentiated coefficient $e^{\beta 1}$ represent the RR associated with a change in covid:

If $\beta 1$ is positive, higher incidences are observed post COVID-19

If $\beta 1$ is negative, lower incidences are observed post COVID-19

We used the function *summ* from the package *jtools* in R to convert the log-scale coefficients into RR, including the confidence intervals and p-values to make the interpretation of the changes pre/post COVID-19 more intuitive. The confidence intervals in the function are computed as:

$$CI= [e^{\beta 1-1.96*SE}, e^{\beta 1+1.96*SE}]$$

Results

Table S1 Average population and number of RTI, RSV-coded and RSV-confirmed admissions among patients 18 years of age or older stratified by pre and post COVID-19. The average population (and standard deviation) for patients ≥18 years of age is shown for each country and COVID-19 period (pre: 2016/17-2018/19 seasons and post: 2021/22-2022/23 seasons, depending on the country). The total number of RTI admissions analysed per country is shown for each COVID-19 period. The number of RSV-coded and RSV-confirmed admissions, together with the proportion from the RTI admissions is also shown.

Country	Average Population (±SD)		N RTI		N RSV-coded (%)		N RSV-confirmed (%)	
	pre	post	pre	post	pre	post	pre	post
**Denmark**	4,613,978 (±27,809)	4,721,691 (±NA)	133,485	36,223	214, 0.2%	165, 0.5%	751, 0.6%	400, 1.1%
**England**	43,887,516 (±144,368)	44,715,443 (±0)	1,365,52 9	1,548,738	5,510, 0.4%	10,485, 0.7%	NA	NA
**Finland**	4,446,030 (±12,141)	4,525,251 (±13,392)	164,193	99,614	2,395, 1.5%	1,423, 1.4%	3,178, 1.9%	1,663, 1.7%
**Netherlands**	13,823,706 (±106,128)	NA	319,414	NA	3,555, 1.1%	NA	NA	NA
**Scotland**	4,401,524 (±7,247)	4,454,919 (±0)	109,555	75,610	494, 0. 5%	390, 0.5%	880, 0.8%	584, 0.8%
**Spain-Valencia**	371,827 (±50,557)	410,329 (±49,529)	5,719	1,837	16, 0.3%	27, 1.5%	335, 5.9%	65, 3.5%

Internal

Table S2 Numbers and proportions admissions. The average (±SD) number of RTI, RSV-coded and RSV-confirmed admissions is shown for each age group, country, diagnosis group and COVID-19 period (pre: 2016/17-2018/19 seasons and post: 2021/22-2022/23 seasons, depending on the country). The proportion from the RTI admissions for the RSV-coded and RSV-confirmed admissions is also shown.

		Denmark			England		Finland			Netherlands		Scotland			Spain-Valencia		
**Age group**	**COVID-19**	**RTI**	**RSV-coded**	**RSV-confirmed**	**RTI**	**RSV-coded**	**RTI**	**RSV-coded**	**RSV-confirmed**	**RTI**	**RSV-coded**	**RTI**	**RSV-coded**	**RSV-confirmed**	**RTI**	**RSV-coded**	**RSV-confirmed**
18-64	Pre	11,467(10,534,12,400)	23(16,30), 0.2%	63(53,73), 0.5%	216,660 (207,718, 225,602)	898 (556,1240), 0.4%	13,155 (12,521,13,789)	154 (44, 264), 1.2%	210 (54,366), 1.6%	26,896 (20,341,33,451)	293 (250,336), 1.1%	17,600 (17,354,17,846)	77 (69,85), 0.4%	140 (103,177), 0.8%	464 (351,577)	2 (0,4), 0.4%	19 (11, 27), 4.1%
	Post	8,237 (±NA)	51(±NA), 0.6%	108(±NA), 1.3%	271,297 (245,283,297,311)	1,552 (474,2,630), 0.6%	11,576 (10,608,12,544)	154 (77, 231), 1.3%	194 (91,297), 1.7%	NA	NA	11,894 (8,124,15,664)	54 (21,87), 0.5%	82 (24,140), 0.7%	298 (231,365)	3 (0,6), 1%	8 (6,10), 2.7%
65-74	Pre	10,443(9,808,11,078)	15(6,24), 0.1%	64(43,85), 0.6%	121,223 (119,079,123,367)	582 (392,772), 0.5%	11,583 (10,883,12,283)	168 (13, 323), 1.5%	228 (22,434), 2%	28,969 (22,315,35,623)	343 (282,404), 1.2%	10,442 (9,796,11,088)	48 (38,58), 0.5%	94 (85,103), 0.9%	401 (327,475)	0, 0%	19 (12, 26), 4.7%
	Post	7,645 (±NA)	32(±NA), 0.4%	84(±NA), 1.1%	131,280 (128,150,134,410)	1,038 (123,1,953), 0.8%	10,410 (9,312,11,508)	176 (78, 274), 1.7%	202 (82,322), 1.9%	NA	NA	7,279 (5,916,8,642)	38 (1,75), 0.5%	56 (2,110), 0.8%	182 (175,189)	2 (-2,6), 1.1%	3 (0,6), 1.6%
75-84	Pre	12,814(12,232,13,396)	21(11,31), 0.2%	70(44,96), 0.5%	172,590 (169,289,175,891)	694 (382,1,006), 0.4%	15,609 (14,614,16,604)	235 (7, 463), 1.5%	310 (5,615), 2%	31,120 (23,038,39,202)	338 (283,393), 1.1%	14,224 (12,801,15,647)	64 (59,69), 0.4%	114 (99, 129), 0.8%	544 (452,636)	2 (1,3), 0.4%	41 (29, 53), 7.5%
	Post	11,830(±NA)	56 (±NA), 0.5%	129(±NA), 1.1%	193,230 (179,031,207,429)	1,406 (119,2,693), 0.7%	15,102 (12,262,17,942)	198 (78, 318), 1.3%	234 (89,379), 1.5%	NA	NA	10,015 (8,474,11,556)	58 (-4,120), 0.6%	87 (4,170), 0.9%	222 (216,228)	4 (-2,10), 1.8%	12 (2,22), 5.4%
≥ 85	Pre	9,772(9,376,10,168)	12(5,19), 0.1%	53(24,82), 0.5%	172,292 (165,185,179,399)	582 (337,827), 0.3%	14,384 (13,279,15,489)	242 (16, 468), 1.7%	311 (14,608), 2.2%	19,486 (14,383,24,589)	211 (169,253), 1.1%	12,512 (11,305,13,719)	58 (53,63), 0.5%	92 (81,103), 0.7%	497 (371,623)	1 (0,2), 0.2%	33 (17, 49), 6.6%
	Post	8,511 (±NA)	26(±NA), 0.3%	79(±NA), 0.9%	178,562 (166,759,190,365)	1,246 (52, 2440), 0.7%	12,718 (10,508,14,928)	184 (81, 287), 1.4%	202 (71,333), 1.6%	NA	NA	8,616 (7,341,9,891)	46 (-3,95), 0.5%	66 (-10,142), 0.8%	216 (193,239)	4 (0,8), 1.9%	10 (-1,21), 4.6%
**Additional age groups**																	
18-49	Pre	NA	NA	NA	NA	NA	5,700 (5390,6,010)	45 (13,77)	68 (15,121)	NA	NA	8,454 (8,421,8,487)	30 (24,36), 0.4%	51 (34,68), 0.6%	NA	NA	NA
	Post	NA	NA	NA	NA	NA	5,268 (4,383,6,153)	57 (33,81)	77 (43,111)	NA	NA	5,298 (3,271,7,325)	18 (5,31), 0.3%	31 (6,56), 0.6%	NA	NA	NA
50-64	Pre	NA	NA	NA	NA	NA	7,455 (709	108 (30,1	141 (38,2	NA	NA	9,146 (8,9	47 (44,5	88 (67,1	299 (2	2 (0,4),	14 (8,2

Internal

							9,7811)	86)	44)			32,9,360)	0), 0.5%	09), 1%	23,375)	0.7%	0), 4.7%
	Post	NA	NA	NA	NA	NA	6,308 (6,225,6,391)	98 (45,151)	117 (48,186)	NA	NA	6,596 (4,853,8,339)	35 (15,55), 0.5%	51 (18,84), 0.8%	188 (165,211)	3 (0,6), 1.6%	6 (3,9), 3.2%
**Diagnosis groups**																	
Bronchitis &Bronchiolitis	Pre	412(358,466)	18(10,26), 4.4%	17(11,23), 4.1%	9,058(8,881, 9,235)	92 (70,114), 1%	3,362 (3,054,3,670)	329 (78,580), 9.8%	343 (79,607), 10.2%	3,363 (2,522,4,204)	192 (157,227), 5.7%	283 (266,300)	16 (13,19), 5.7%	11 (4,18), 3.9%	129 (102,156)	2 (1,3), 1.6%	18 (7,29), 14%
	Post	292(±NA)	52(±NA), 17.8%	44(±NA), 15.1%	6,204(5,682, 6,726)	168 (96,240), 2.7%	1,021 (922,1,120)	256 (148, 364), 25.1%	246 (132, 360), 24.1%	NA	NA	176 (139,213)	9 (4,14), 5.1%	8 (4,12), 4.5%	34 (25, 43)	3 (3,3), 8.8%	6 (3,9), 17.6%
Unspecified LRTI	Pre	517(341,693)	2(±NA), 0.4%	7(4,10), 1.4%	175,238(173,822,176,654)	599 (379,819), 0.3%	1,117 (1,046,1,188)	1 (-1,3), 0.1%	6 (3,9), 0.5%	1,901 (1,317,2,485)	67 (43,91), 3.5%	20,585 (19,611,21,559)	86 (73,99), 0.4%	164 (132,196), 0.8%	159 (46,272)	NA	20 (8,32), 12.6%
	Post	297(±NA)	NA	4(±NA), 1.3%	122,066(114,133,129,999)	1,025 (383,1,667), 0.8%	524 (511,537)	0 (0,0), 0%	5 (4,6), 1%	NA	NA	11,752 (10,245,13,259)	64 (20,108), 0.5%	106 (35, 177), 0.9%	44 (39, 49)	1 (±0), 2.3%	2 (1,3), 4.5%
URTI	Pre	1,616 (1,499,1,733)	NA	5 (4,6), 0.3%	49,344 (48,288,50,400)	127 (78,176), 0.3%	3,542 (3,392,3,692)	3 (0,6), 0.1%	32 (9,55), 0.9%	12,519 (9,538,15,500)	208 (172,244), 1.7%	3,812 (3,762,3,862)	22 (18,26), 0.6%	32 (15,49), 0.8%	30 (8,52)	NA	7 (2,12), 23.3%
	Post	1,450 (±NA)	NA	13(±NA), 0.9%	38,688 (33,177,44,199)	155 (82,228), 0.4%	1,464 (1,311,1,617)	2 (0,4), 0.1%	18 (7,29), 1.2%	NA	NA	2,204 (1,780,2,628)	10 (8,12), 0.5%	10 (5,15), 0.5%	2 (1,3)	NA	1 (±0), 50%
Pneumonia&Influenza	Pre	41,596 (39,683,43,509)	49 (27, 71), 0.1%	220 (153,287), 0.5%	440,098 (435,069,445,127)	1,212 (837,1587), 0.3%	45,443 (43,169,47,717)	354 (80,628), 0.8%	556 (113, 999), 1.2%	87,746 (69, 781,105,711)	652 (600,704), 0.7%	30,099 (28,443,31,755)	119 (117,121), 0.4%	232 (229,235), 0.8%	831 (628,1034)	5 (5,5), 0.6%	26 (18, 34), 3.1%
	Post	33,835 (± NA)	111 (± NA), 0.3%	328(±NA), 1%	334,450 (300,266,368,634)	2,055 (613,3,497), 0.6%	24,347 (23,874,24,820)	356 (207, 505), 1.5%	445 (250, 640), 1.8%	NA	NA	23,661 (19,622,27,700)	112 (26, 198), 0.5%	169 (46, 292), 0.7%	514 (487,541)	10 (5,15), 1.9%	13 (7,19), 2.5%
SARSCOV-2	Post	NA	NA	NA	165,462 (143,401,187,523)	60 (13,107), 0%	16,491 (13,517,19,465)	3 (2,4), 0%	18 (7,29), 0.1%	NA	NA	13,026 (8, 287,17,765)	8 (8,8), 0.1%	18 (10,26), 0.1%	144 (53,235)	1 (±0), 0.7%	1 (±0), 0.7%
> 1 Diagnosis	Pre	NA	NA	NA	8,406 (7,912,8,900)	105 (93,117), 1.2%	1,266 (1,155,1,377)	111 (23,199), 8.8%	119 (27,211), 9.4%	943 (791,1, 095)	67 (59,75), 7.1%	NA	NA	NA	23 (11, 35)	NA	3 (1,5), 15%
	Post	NA	NA	NA	106,118 (81, 582,130,654)	398 (96,700), 0.4%	5,960 (5,730,6,190)	94 (53,135), 1.6%	98 (53,143), 1.6%	NA	NA	NA	NA	NA	85 (35, 135)	2 (2,2), 2.4%	1 (1,1), 1.2%

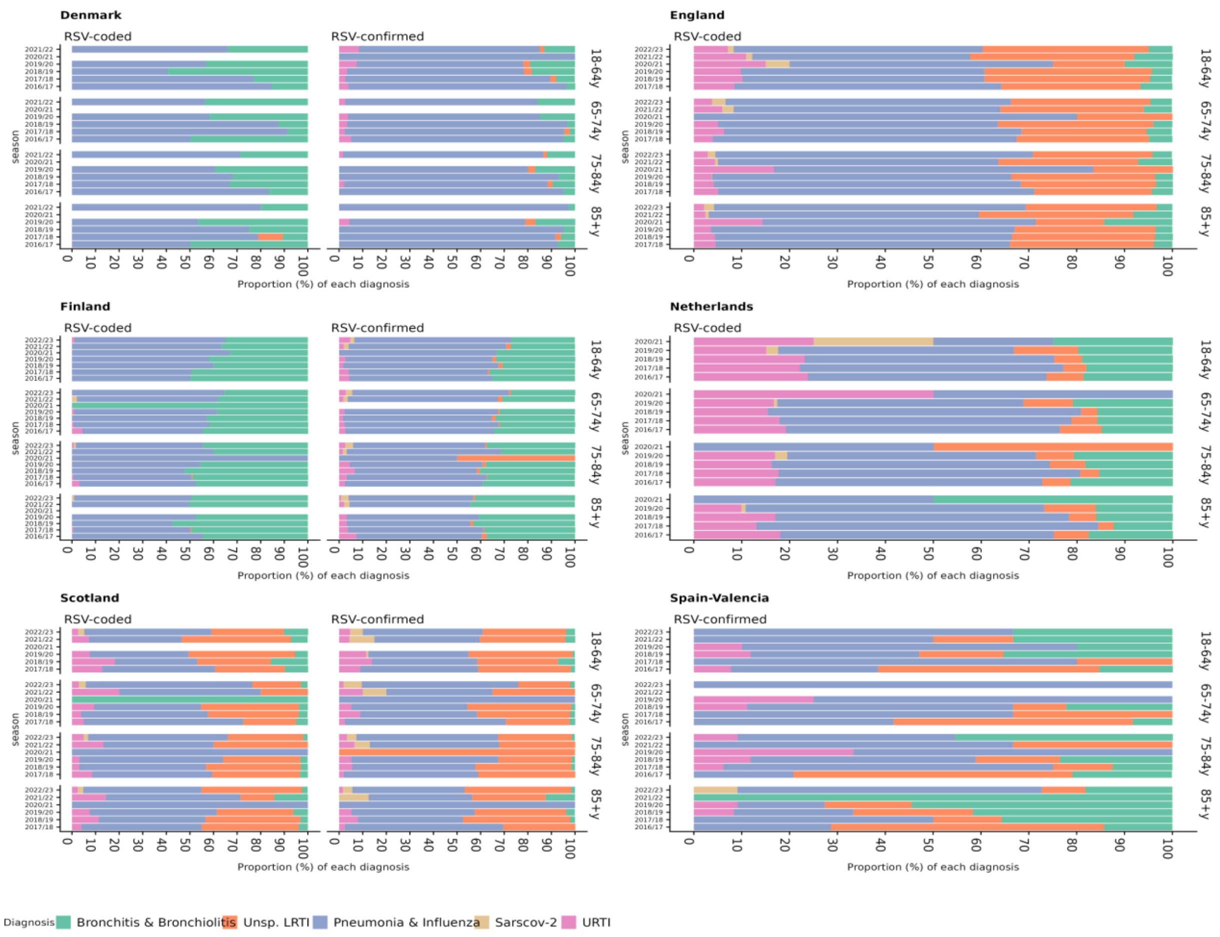

Figure S1 Diagnosis groups among RSV-hospitalisations. Each RSV-coded or RSV-confirmed hospitalisation was classified by the following diagnosis groups (Bronchitis & Bronchiolitis, Unspecified LRTI, Pneumonia & Influenza, SARSCOV-2 or URTI). The proportion in percentage (x-axis) of each of these diagnosis groups per country, season (y-axis), age group and hospitalisation type are shown.

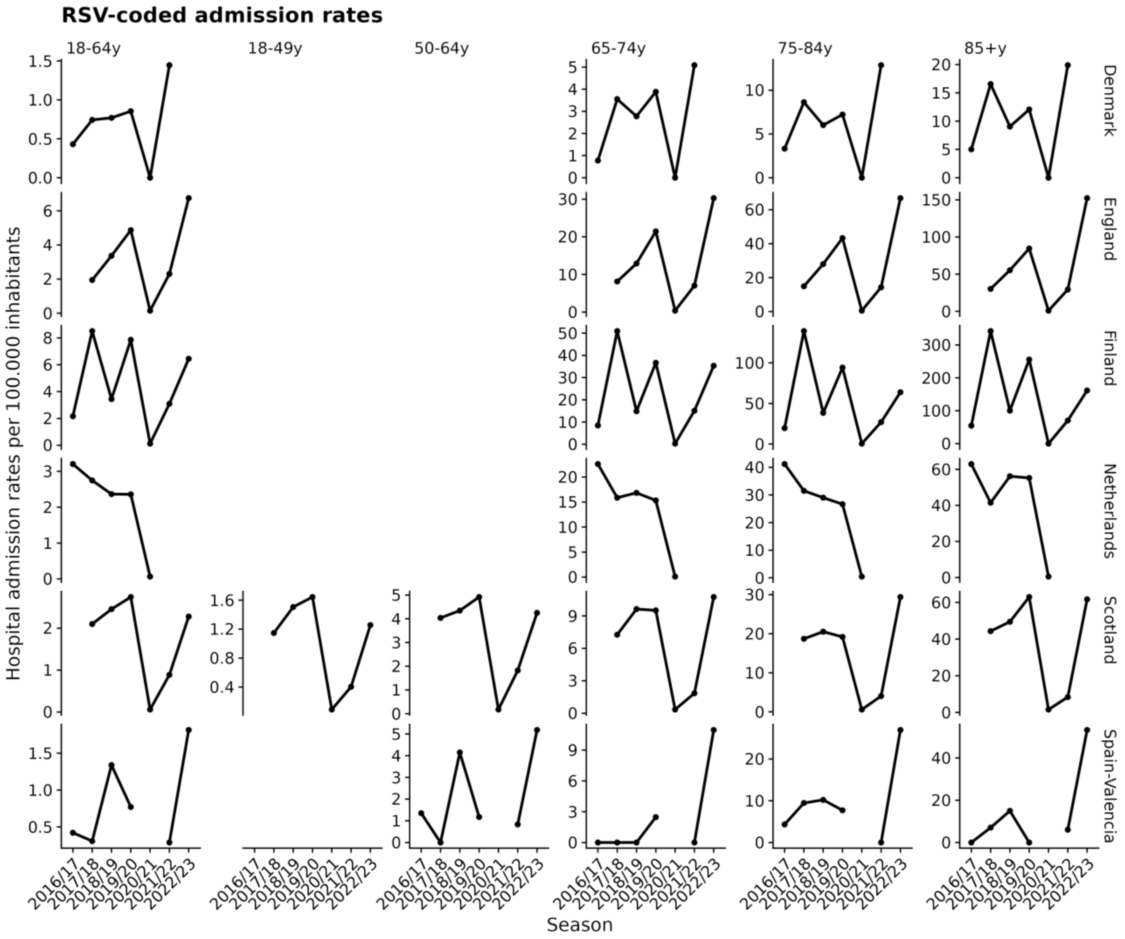

Figure S2 RSV-coded hospitalisation admission rates in patients 18 years of age and above. The RSV-coded hospital admissions rates per 100,000 persons-year (y-axis) and season (x-axis) are shown for each country, age group (vertical subpanels) and hospitalisation type. Note that each panel has a different y-axis.

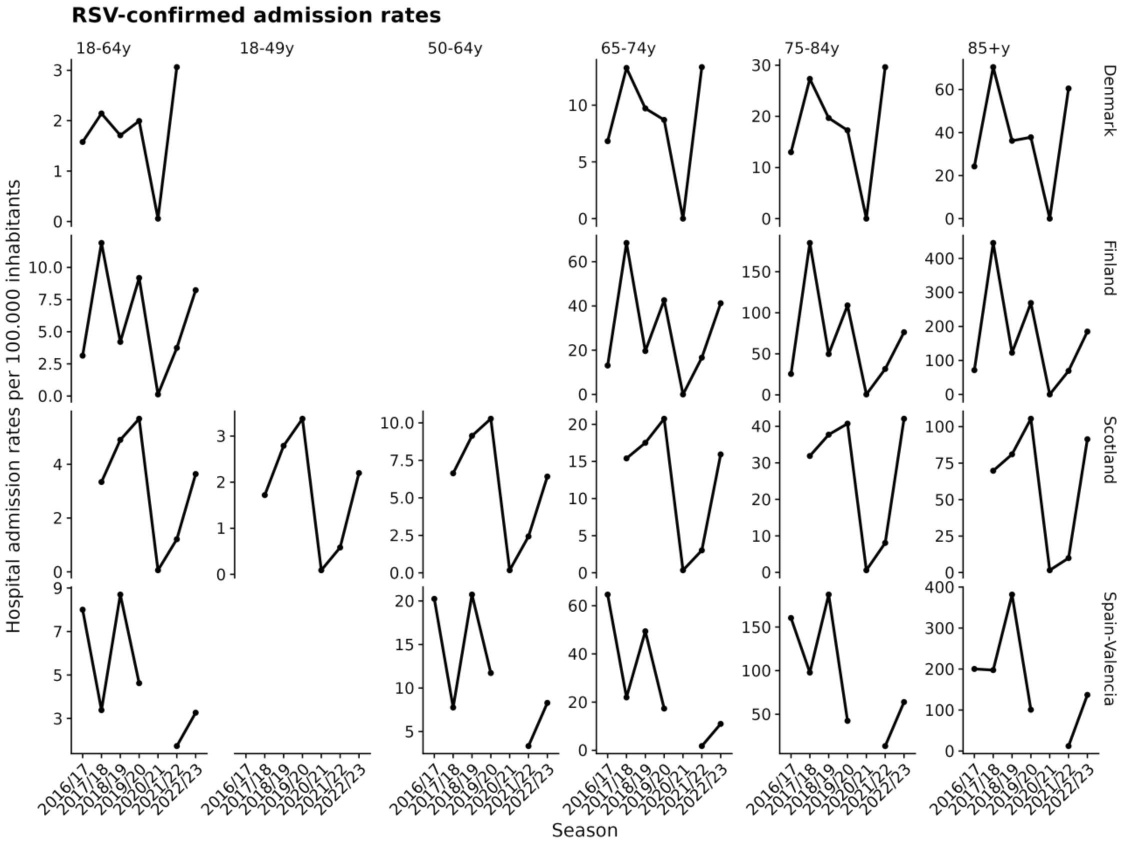

Figure S3 RSV-confirmed hospitalisation admission rates in patients 18 years of age and above. The RSV-confirmed hospital admissions rates per 100,000 persons-year (y-axis) and season (x-axis) are shown for each country, age group (vertical subpanels) and hospitalisation type. Note that each panel has a different y-axis.

Table S3 RSV-coded admission rates (95%CI) per season, country and age group. The RSV-coded admission rate for adults ≥18 years per 100,000 persons-year together with the 95%CI is shown per age group, country and season.

Surv. year	Age group	Denmark	England	Finland	Netherlands	Scotland	Spain-Valencia
2016/17	18-64y	0.4 [0.2-0.7]	NA	2.2 [1.7-2.7]	3.2 [2.9-3.6]	NA	0.4 [0-2.3]
2017/18		0.7 [0.5-1.1]	1.9 [1.8-2.1]	8.5 [7.5-9.6]	2.7 [2.4-3.1]	2.1 [1.6-2.6]	0.3 [0-1.7]
2018/19		0.8 [0.5-1.1]	3.4 [3.2-3.6]	3.4 [2.8-4.1]	2.4 [2.1-2.7]	2.5 [2-3]	1.3 [0.4-3.4]
2019/20		0.9 [0.6-1.2]	4.9 [4.6-5.1]	7.9 [6.9-8.9]	2.4 [2.1-2.7]	2.7 [2.2-3.4]	0.8 [0.1-2.8]
2020/21		0 [0-0.1]	0.1 [0.1-0.2]	0.1 [0-0.3]	0.1 [0-0.1]	0.1 [0-0.2]	NA
2021/22		1.4 [1.1-1.9]	2.3 [2.1-2.5]	3.1 [2.5-3.8]	NA	0.9 [0.6-1.3]	0.3 [0-1.6]
2022/23		NA	6.7 [6.5-7]	6.4 [5.6-7.4]		2.3 [1.8-2.8]	1.8 [0.6-4.2]
2016/17	18-49y	NA				NA	NA
2017/18						1.1 [0.7-1.7]	
2018/19						1.5 [1-2.1]	

2019/20						1.6 [1.2-2.3]	
2020/21						0.1 [0-0.3]	
2021/22						0.4 [0.2-0.8]	
2022/23						1.3 [0.8-1.8]	
2016/17						NA	1.3 [0-7.5]
2017/18						4 [2.9-5.4]	0 [0-3.6]
2018/19						4.3 [3.2-5.7]	4.1 [1.1-10.6]
2019/20	50-64y	NA				4.9 [3.7-6.4]	1.2 [0-6.5]
2020/21						0.2 [0-0.6]	NA
2021/22						1.8 [1.1-2.8]	0.8 [0-4.6]
2022/23						4.3 [3.1-5.6]	5.2 [1.7-12.1]
2016/17		0.8 [0.3-1.8]	NA	8.5 [6.4-11]	22.6 [20.5-24.9]	NA	0 [0-10.4]
2017/18		3.6 [2.3-5.3]	8.1 [7.4-8.9]	50.9 [45.7-56.6]	15.9 [14.1-17.8]	7.3 [5.2-9.9]	0 [0-7.4]
2018/19		2.8 [1.6-4.4]	12.9 [12-13.9]	14.9 [12.1-18]	16.8 [15-18.8]	9.6 [7.3-12.5]	0 [0-7.9]
2019/20	65-74y	3.9 [2.5-5.7]	21.4 [20.3-22.7]	36.6 [32.3-41.4]	15.3 [13.6-17.2]	9.5 [7.2-12.4]	2.5 [0.1-13.8]
2020/21		0 [0-0.6]	0.4 [0.2-0.6]	0.3 [0-1]	0.1 [0-0.4]	0.3 [0-1.2]	NA
2021/22		5.1 [3.5-7.2]	7 [6.3-7.8]	15.1 [12.3-18.2]	NA	1.8 [0.9-3.3]	0 [0-6.5]
2022/23		NA	30.3 [28.9-31.8]	35.3 [31-40]	NA	10.7 [8.3-13.7]	11 [3.6-25.6]
2016/17		3.3 [1.7-5.9]	NA	19.6 [15.3-24.8]	41.2 [37.2-45.4]	NA	4.3 [0.1-24.1]
2017/18		8.6 [5.8-12.3]	14.9 [13.6-16.3]	139.2 [127.2-152]	31.5 [28.1-35.2]	18.7 [14.3-24]	9.5 [2-27.7]
2018/19		6 [3.8-9.1]	28 [26.2-29.9]	38.4 [32.3-45.3]	29 [25.8-32.5]	20.5 [15.9-26]	10.2 [2.1-29.8]
2019/20	75-84y	7.2 [4.8-10.4]	43.3 [41.1-45.6]	94.2 [84.6-104.6]	26.7 [23.6-29.9]	19.2 [14.8-24.5]	7.7 [0.9-27.9]
2020/21		0 [0-0.9]	0.5 [0.3-0.8]	0.5 [0.1-1.8]	0.5 [0.1-1.1]	0.6 [0.1-2.1]	NA
2021/22		12.9 [9.7-16.7]	14.3 [13.1-15.6]	27 [22.3-32.5]	NA	4 [2.2-6.8]	0 [0-10]
2022/23		NA	66.8 [64.1-69.6]	63.8 [56.6-71.7]	NA	29.4 [24-35.7]	27 [11.6-53.1]
2016/17		5 [1.8-10.9]	NA	54.6 [43.2-68.1]	62.9 [55.3-71.4]	NA	0 [0-36.9]
2017/18		16.6 [10.1-25.6]	30.2 [27.3-33.3]	342.1 [312.7-373.4]	41.5 [35.4-48.3]	44.3 [33.3-57.8]	7.1 [0.2-39.3]
2018/19		9 [4.5-16.2]	55.3 [51.4-59.4]	100.3 [84.8-117.8]	56.2 [49.1-64]	49.4 [37.8-63.5]	15 [1.8-54.1]
2019/20	≥85y	12.1 [6.8-19.9]	84.5 [79.7-89.4]	255.6 [230.7-282.4]	55.2 [48.3-62.9]	62.9 [49.9-78.3]	0 [0-31]
2020/21		0 [0-2.9]	0.9 [0.4-1.5]	0 [0-2.4]	0.5 [0.1-1.7]	1.6 [0.2-5.6]	NA
2021/22		19.9 [13-29.2]	29.3 [26.5-32.3]	70.4 [57.9-84.8]	NA	8.4 [4.2-15]	6.1 [0.2-33.8]
2022/23		NA	152.3 [145.8-159]	161.5 [142.4-182.5]	NA	61.7 [49-76.7]	53.3 [21.4-109.7]

Table S4 RSV-confirmed admission rates (95%CI) per season, country and age group. The RSV-confirmed admission rate for adults ≥18 years per 100,000 persons-year together with the 95%CI is shown per age group, country and season.

Surv. year	Age group	Denmark	Finland	Scotland	Spain-Valencia
2016/17	18-64y	1.6 [1.2-2.1]	3.1 [2.6-3.8]	NA	8 [4.8-12.5]

2017/18		2.1 [1.7-2.7]	11.9 [10.8-13.1]	3.3 [2.8-4]	3.4 [1.7-6.1]
2018/19		1.7 [1.3-2.2]	4.2 [3.5-5]	4.9 [4.2-5.7]	8.7 [5.7-12.7]
2019/20		2 [1.6-2.5]	9.2 [8.2-10.3]	5.7 [4.9-6.6]	4.6 [2.4-8.1]
2020/21		0.1 [0-0.2]	0.1 [0-0.3]	0.1 [0-0.2]	NA
2021/22		3.1 [2.5-3.7]	3.7 [3.1-4.5]	1.2 [0.9-1.6]	1.7 [0.6-3.8]
2022/23		NA	8.2 [7.3-9.3]	3.6 [3-4.3]	3.3 [1.5-6.2]
2016/17	18-49y	NA	NA	NA	NA
2017/18				1.7 [1.2-2.4]	
2018/19				2.8 [2.1-3.6]	
2019/20				3.4 [2.7-4.2]	
2020/21				0.1 [0-0.3]	
2021/22				0.6 [0.3-1]	
2022/23				2.2 [1.6-2.9]	
2016/17	50-64y	NA	NA	NA	20.2 [11.3-33.4]
2017/18				6.6 [5.2-8.3]	7.8 [3.4-15.3]
2018/19				9.1 [7.5-11.1]	20.7 [12.7-32]
2019/20				10.3 [8.5-12.3]	11.7 [5.6-21.6]
2020/21				0.2 [0-0.6]	NA
2021/22				2.4 [1.6-3.5]	3.3 [0.9-8.5]
2022/23				6.4 [5-8.1]	8.3 [3.6-16.3]
2016/17	65-74y	6.8 [5-9.2]	13.1 [10.4-16.2]	NA	64.6 [41-97]
2017/18		13.3 [10.6-16.4]	68.5 [62.4-75]	15.4 [12.3-19]	22 [11-39.3]
2018/19		9.7 [7.5-12.4]	19.6 [16.5-23.2]	17.5 [14.2-21.3]	49.4 [31.3-74.1]
2019/20		8.7 [6.6-11.3]	42.6 [37.9-47.7]	20.8 [17.2-24.8]	17.3 [7-35.7]
2020/21		0 [0-0.6]	0 [0-0.5]	0.3 [0-1.2]	NA
2021/22		13.3 [10.6-16.5]	16.6 [13.8-19.9]	3 [1.8-4.8]	1.8 [0-9.8]
2022/23		NA	41.2 [36.6-46.3]	16 [12.9-19.5]	11 [3.6-25.6]
2016/17	75-84y	13 [9.4-17.5]	25.5 [20.5-31.3]	NA	160.4 [112.9-221]
2017/18		27.3 [22.1-33.4]	185 [171.2-199.7]	31.9 [26.1-38.6]	97.8 [66.5-138.8]
2018/19		19.7 [15.4-24.8]	49.7 [42.7-57.5]	37.7 [31.4-44.9]	187.2 [141-243.7]
2019/20		17.3 [13.4-21.9]	108.9 [98.6-120.1]	40.7 [34.2-48.1]	42.5 [21.2-76]
2020/21		0 [0-0.9]	0.5 [0.1-1.8]	0.6 [0.1-2.1]	NA
2021/22		29.6 [24.7-35.2]	31.5 [26.4-37.4]	8.1 [5.4-11.7]	13.6 [4.4-31.6]
2022/23		NA	76.3 [68.4-84.9]	42.1 [35.5-49.5]	64 [38.5-100]
2016/17	≥85y	24.2 [16.2-34.8]	71.4 [58.2-86.7]	NA	200.3 [122.3-309.3]
2017/18		70.4 [56.2-87]	445.4 [411.8-481]	69.8 [55.7-86.3]	197.5 [131.2-285.4]
2018/19		36.2 [26.3-48.6]	122.6 [105.4-141.8]	81.1 [65.9-98.6]	381.7 [284.2-501.9]
2019/20		37.8 [27.8-50.3]	268.9 [243.3-296.3]	105.4 [88.3-124.9]	100.8 [52.1-176]
2020/21		0 [0-2.9]	0 [0-2.4]	1.6 [0.2-5.6]	NA
2021/22		60.5 [47.9-75.4]	69.1 [56.8-83.4]	9.9 [5.3-16.9]	12.1 [1.5-43.8]
2022/23		NA	184.8 [164.3-207.1]	91.4 [75.8-109.3]	136.9 [81.2-216.4]

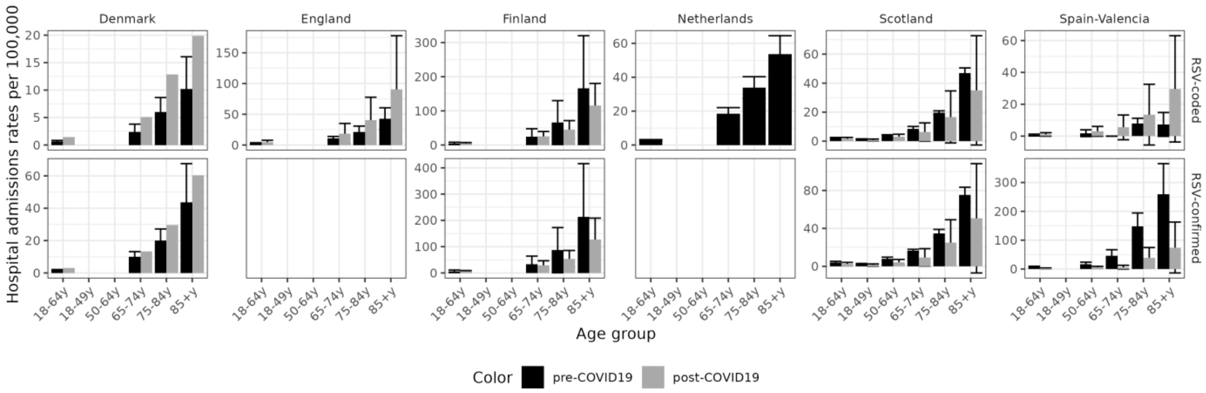

Figure S4 Average hospital admission rates pre and post COVID-19. The average (±SD) of the hospital admission rates (y-axis) pre COVID-19 seasons: 2016/17-2018/19 (black) and post COVID-19 seasons: 2021/22-2022(23 (grey) are shown for each country (horizontal subpanels) and age group (x-axis) for both RSV-coded and RSV-confirmed admissions.

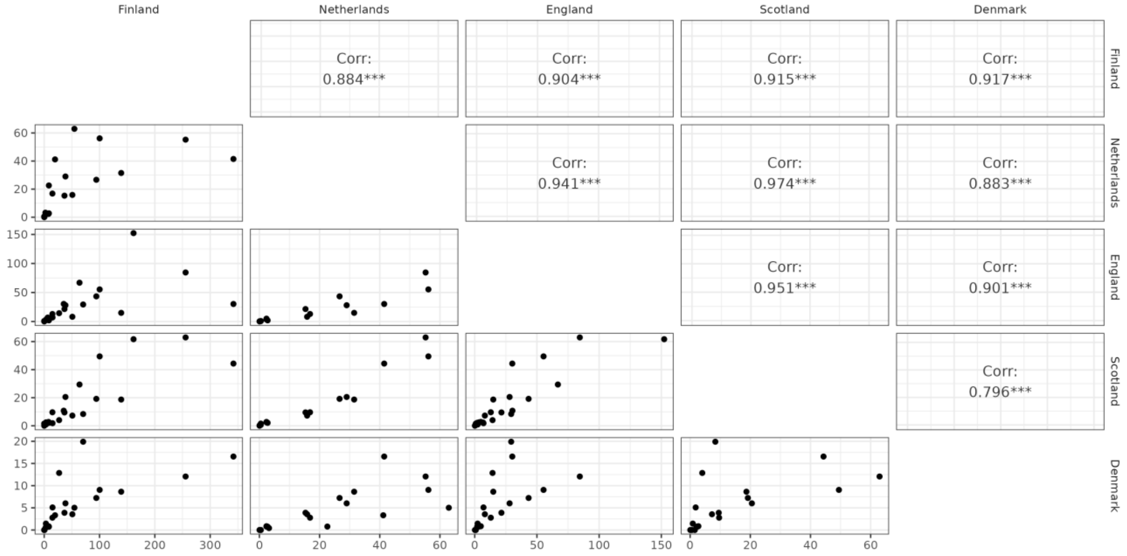

Figure S5 Pairwise country RSV-coded hospital admission rates correlation factors. For each pair of countries with available RSV-coded admission rates (vertical and horizontal panels) the admission rates in each season and age group are plotted against each other. Pairwise spearman correlation factors are indicated at the top right for each pair of countries as indicated by the panels. Stars indicate that the correlation among both hospitalisation datasets is significant. Spain-Valencia was not included in the analysis due to the low number of diagnoses codes recorded at discharge (3).

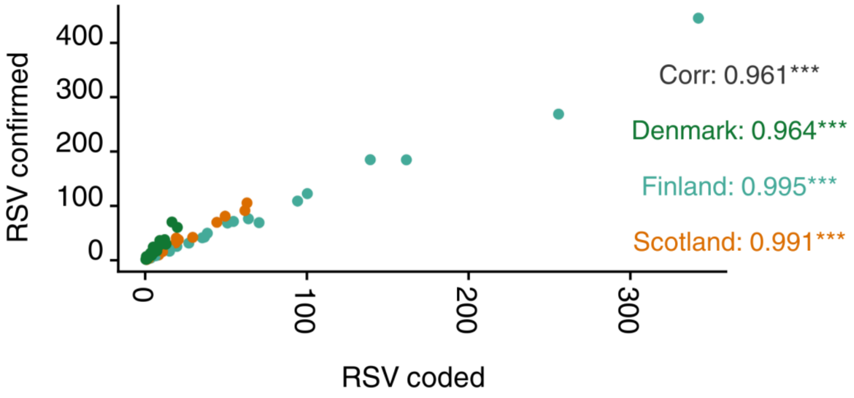

Figure S6 RSV-coded versus RSV-confirmed hospital admission rates correlation factors. For each season and age group, the RSV-coded (x-axis) and RSV-confirmed (y-axis) hospitalisation rate are plotted against each other in countries with both types of data available (different colours). Pairwise spearman correlation factors are indicated at the top right, overall and for each country. Stars indicate that the correlation among both hospitalisation datasets is significant.

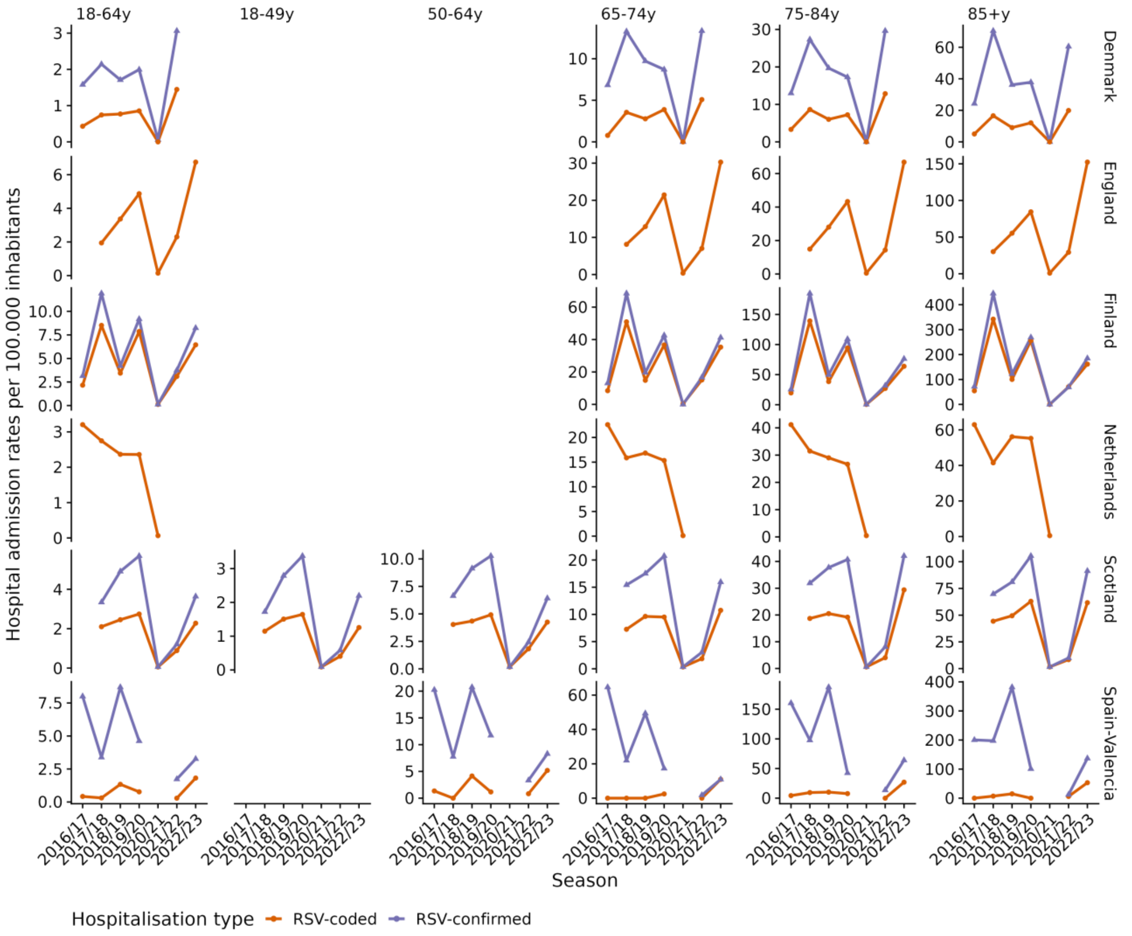

Figure S7 RSV-coded and confirmed admissions per 100,000 person-years. RSV-coded (orange) and RSV-confirmed (violet) admission rates are shown together on the same graph per country, age group and season.

Internal

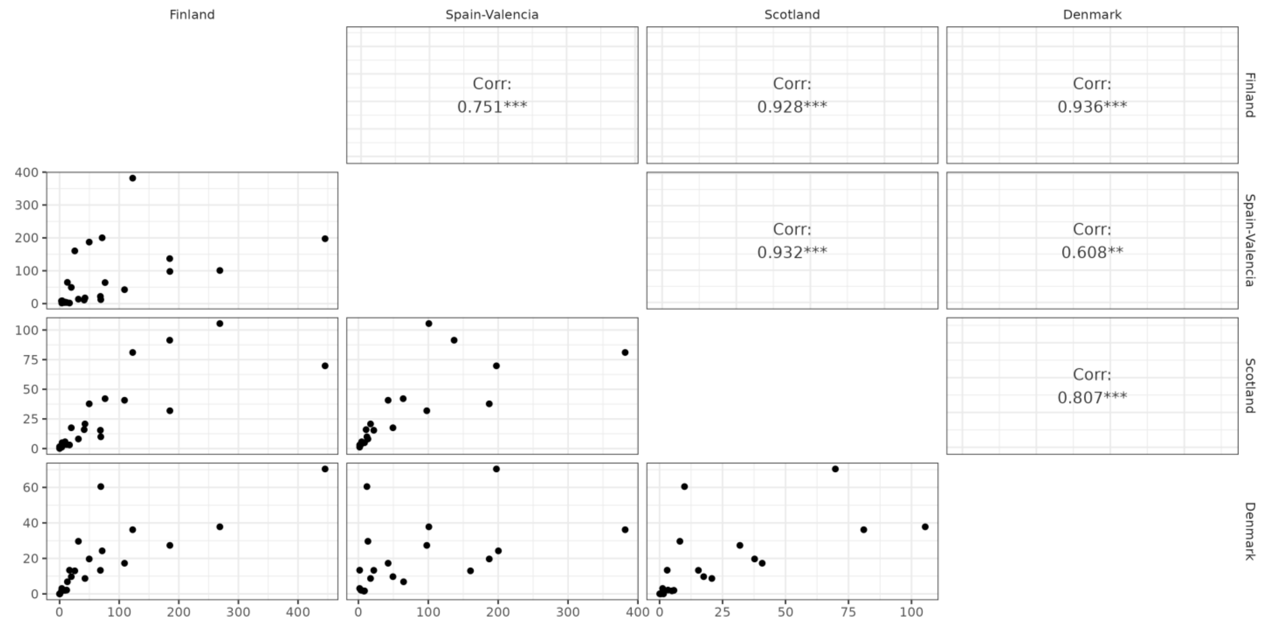

Figure S8 Pairwise country RSV-confirmed hospital admission rates correlation factors. For each pair of countries with available RSV-confirmed admission rates (vertical and horizontal panels) the admission rates in each season and age group are plotted against each other. Pairwise Spearman correlation factors are indicated at the top right for each pair of countries as indicated by the panels. Stars indicate that the correlation among both hospitalisation datasets is significant.

Table S5 RSV-coded average pre/post COVID-19 hospital admissions by country and age group. The table shows the average (± standard deviation) of RSV-coded hospital admission rates pre COVID-19 (2016/17-2018/19, AvgIRPre) and of the pre-COVID-19 seasons including season 2019/20 (AvgIRPre incl.2019/20). The average (± standard deviation) of RSV-coded hospital admission rates post COVID-19 is shown in the AvgIRPost column.

Country	Age group	AvgIRPre±SD	AvgIRPre±SD incl. 2019/20	AvgIRPost±SD
Denmark	18-64	0.6+/-0.2	0.7+/-0.2	1.4+/-NA
	65-74	2.4+/-1.4	2.7+/-1.4	5.1+/-NA
	75-84	6+/-2.7	6.3+/-2.3	12.9+/-NA
	≥85	10.2+/-5.9	10.7+/-4.9	19.9+/-NA
England	18-64	2.7+/-1	3.4+/-1.5	4.5+/-3.1
	65-74	10.5+/-3.4	14.2+/-6.7	18.7+/-16.4
	75-84	21.4+/-9.3	28.7+/-14.2	40.6+/-37.1
	≥85	42.7+/-17.8	56.7+/-27.2	90.8+/-87
Finland	18-64	4.7+/-3.4	5.5+/-3.2	4.8+/-2.4
	65-74	24.7+/-22.9	27.7+/-19.6	25.2+/-14.3
	75-84	65.7+/-64.3	72.9+/-54.4	45.4+/-26
	≥85	165.6+/-154.5	188.1+/-133.9	116+/-64.4
Netherlands	18-64	2.8+/-0.4	2.7+/-0.4	NA
	65-74	18.4+/-3.6	17.7+/-3.3	

	75-84	33.9+/-6.4	32.1+/-6.4	
	≥85	53.5+/-11	54+/-9	
Scotland	18-64	2.3+/-0.3	2.4+/-0.3	1.6+/-1
	65-74	8.4+/-1.7	8.8+/-1.3	6.3+/-6.3
	75-84	19.6+/-1.3	19.5+/-0.9	16.7+/-17.9
	≥85	46.9+/-3.6	52.2+/-9.6	35+/-37.7
Spain-Valencia	18-64	0.7+/-0.6	0.7+/-0.5	1.1+/-1.1
	65-74	0+/-0	0.6+/-1.2	5.5+/-7.8
	75-84	8+/-3.2	7.9+/-2.6	13.5+/-19.1
	≥85	7.3+/-7.5	5.5+/-7.1	29.7+/-33.4

Table S6 RSV-confirmed average pre/post COVID-19 hospital admissions by country and age group. The table shows the average (± standard deviation) of RSV-confirmed hospital admission rates pre COVID-19 (2016/17-2018/19, AvgIRPre) and of the pre-COVID-19 seasons including season 2019/20 (AvgIRPre incl.2019/20). The average (± standard deviation) of RSV-confirmed hospital admission rates post COVID-19 is shown in the AvgIRPost column.

**Country**	**Age group**	**AvgIRPre±SD**	**AvgIRPre±SD incl. 2019/20**	**AvgIRPost±SD**
Denmark	18-64	1.8+/-0.3	1.9+/-0.3	3.1+/-NA
	65-74	9.9+/-3.2	9.6+/-2.7	13.3+/-NA
	75-84	20+/-7.2	19.3+/-6	29.6+/-NA
	≥85	43.6+/-23.9	42.1+/-19.8	60.5+/-NA
Finland	18-64	6.4+/-4.8	7.1+/-4.1	6+/-3.2
	65-74	33.7+/-30.3	35.9+/-25.1	28.9+/-17.4
	75-84	86.7+/-86	92.3+/-71.1	53.9+/-31.6
	≥85	213.1+/-202.8	227.1+/-167.9	127+/-81.8
Scotland	18-64	4.1+/-1.1	4.6+/-1.2	2.4+/-1.7
	65-74	16.5+/-1.5	17.9+/-2.7	9.5+/-9.1
	75-84	34.8+/-4.1	36.8+/-4.5	25.1+/-24
	≥85	75.4+/-8	85.4+/-18.2	50.6+/-57.6
Spain-Valencia	18-64	6.7+/-2.9	6.2+/-2.6	2.5+/-1.1
	65-74	45.3+/-21.6	38.3+/-22.5	6.4+/-6.5
	75-84	148.5+/-45.9	122+/-64.9	38.8+/-35.7
	≥85	259.8+/-105.6	220.1+/-117.3	74.5+/-88.3

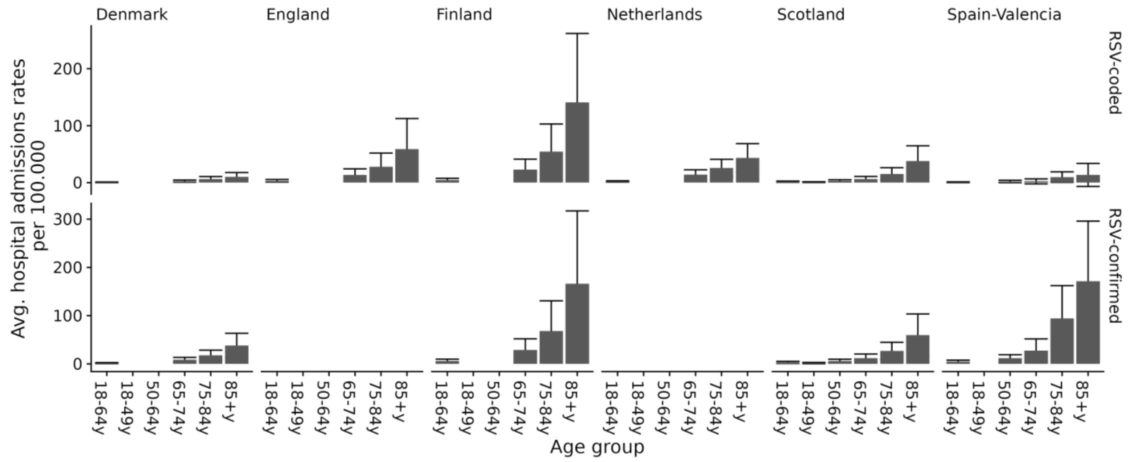

Figure S9 Average hospital admission rates over the whole study period. The average (±SD) of the hospital admission rates over the whole study period (y-axis) are shown for each country (horizontal subpanels, different colours) and age group (x-axis) for both RSV-coded and RSV-confirmed admissions.

Table S7 Comparison of RSV-Coded Hospitalization Incidence Rates Pre- and Post-COVID-19 across Countries and Age Groups. This table presents the risk ratios (RR) comparing RSV-coded hospitalization incidence rates before (2016/17–2018/19) and after COVID-19 across different countries and age groups. It provides estimates of how hospitalization rates have changed post-COVID-19, along with confidence intervals (CI) and p-values to assess statistical significance.

- **RR (Risk Ratio):** A value above 1 indicates an increased incidence post-COVID-19, while a value below 1 suggests a decline.
- **Increase/Decrease:** Represents the percentage change, calculated as *1 – RR* (if RR > 1) or *RR – 1* (if RR < 1), then multiplied by 100. A negative value indicates a decline.
- **Significant changes (p-value < 0.05):** Marked in blue to highlight statistically meaningful differences.

Country	Age group	RR	post-COVID-19 Increase/Decrease (%)	p-value
Denmark	18-64	-	-	-
	65-74	2.57 [0.84-7.85]	157	0.098
	75-84	1.64 [0.82-3.29]	64	0.159
	≥85	1.68 [0.98-2.9]	68	0.059
England	18-64	1.48 [0.55-3.94]	48	0.434
	65-74	1.35 [0.86-2.1]	35	0.188
	75-84	1.34 [0.98-1.81]	34	0.063
	**≥85**	**1.61 [1.3-1.98]**	**61**	**<0.001**
Finland	18-64	0.91 [0.41-1.99]	-9	0.807
	65-74	0.9 [0.65-1.26]	-10	0.555
	**75-84**	**0.52 [0.41-0.66]**	**-48**	**<0.001**
	**≥85**	**0.57 [0.49-0.66]**	**-43**	**<0.001**
Scotland	18-64	0.5 [0.1-2.48]	-50	0.397
	65-74	0.63 [0.31-1.29]	-37	0.206

	75-84	0.83 [0.54-1.28]	-17	0.398
	**≥85**	**0.63 [0.48-0.84]**	**-37**	**<0.001**

Table S8 Comparison of RSV-Coded Hospitalization Incidence Rates Pre- and Post-COVID-19 across Countries and Age Groups including season 2019/20. This table presents the risk ratios (RR) comparing RSV-coded hospitalization incidence rates before (2016/17–2019/20) and after COVID-19 across different countries and age groups. It provides estimates of how hospitalization rates have changed post-COVID-19, along with confidence intervals (CI) and p-values to assess statistical significance.

- **RR (Risk Ratio):** A value above 1 indicates an increased incidence post-COVID-19, while a value below 1 suggests a decline.
- **post-COVID-19 Increase/Decrease (%):** Represents the percentage change, calculated as *1 – RR* (if RR > 1) or *RR – 1* (if RR < 1), then multiplied by 100. A negative value indicates a decline.
- **AvgIRPre:** The average (± standard deviation) of hospital admission rates pre COVID-19.
- **AvgIRPost:** The average (± standard deviation) of hospital admission rates post COVID-19.
- **Significant changes (p-value < 0.05):** Marked in blue to highlight statistically meaningful differences.

Country	Age group	RR	post-COVID-19 Increase/Decrease (%)	p-value
Denmark	18-64	-	-	-
	65-74	3.08 [0.89-10.65]	208	0.075
	75-84	1.69 [0.81-3.55]	69	0.162
	≥85	1.75 [0.99-3.12]	75	0.055
England	18-64	1.97 [0.59-6.54]	97	0.269
	**65-74**	**1.84 [1.07-3.16]**	**84**	**0.029**
	**75-84**	**1.77 [1.22-2.58]**	**77**	**<0.001**
	**≥85**	**2.11 [1.63-2.73]**	**111**	**<0.001**
Finland	18-64	1.05 [0.45-2.45]	5	0,914
	65-74	1 [0.7-1.44]	0	0,98
	**75-84**	**0.57 [0.45-0.74]**	**-43**	**<0.001**
	**≥85**	**0.64 [0.55-0.75]**	**-36**	**<0.001**
Scotland	18-64	0.5 [0.09-2.73]	-50	0.424
	65-74	0.66 [0.3-1.41]	-34	0.281
	75-84	0.82 [0.52-1.31]	-18	0.412
	**≥85**	**0.69 [0.51-0.95]**	**-31**	**0.021**

Table S9 Comparison of RSV-Confirmed Hospitalization Incidence Rates Pre- and Post-COVID-19 across Countries and Age Groups. This table presents the risk ratios (RR) comparing RSV-confirmed hospitalization incidence rates before (2016/17–2018/19) and after COVID-19 across different countries and age groups. It provides estimates of how hospitalization rates have changed post-COVID-19, along with confidence intervals (CI) and p-values to assess statistical significance.

- **RR (Risk Ratio):** A value above 1 indicates an increased incidence post-COVID-19, while a value below 1 suggests a decline.
- **post-COVID-19 Increase/Decrease (%):** Represents the percentage change, calculated as *1 – RR* (if RR > 1) or *RR – 1* (if RR < 1), then multiplied by 100. A negative value indicates a decline.
- **AvgIRPre:** The average (± standard deviation) of hospital admission rates pre-COVID-19.
- **AvgIRPost:** The average (± standard deviation) of hospital admission rates post-COVID-19.
- **Significant changes (p-value < 0.05):** Marked in blue to highlight statistically meaningful differences.

Country	Age group	RR	post-COVID-19 Increase/Decrease (%)	p-value
Denmark	18-64	2.23 [0.5-9.97]	123	0.293
	65-74	1.43 [0.74-2.76]	43	0.285
	75-84	1.2 [0.77-1.87]	20	0.424
	≥85	1.28 [0.94-1.74]	28	0.115
Finland	18-64	0.93 [0.44-1.96]	-7	0.839
	65-74	0.82 [0.6-1.14]	-18	0.245
	**75-84**	**0.52 [0.41-0.65]**	**-48**	**<0.001**
	**≥85**	**0.55 [0.47-0.63]**	**-45**	**<0.001**
Scotland	18-64	0.57 [0.17-1.95]	-43	0,372
	**65-74y**	**0.54 [0.3-0.96]**	**-46**	**0,034**
	75-84	0.7 [0.48-1]	-30	0,052
	**≥85**	**0.62 [0.48-0.8]**	**-38**	**<0.001**
Spain-Valencia	**18-64**	**0.29 [0.1-0.86]**	**-71**	**0.025**
	**65-74**	**0.11 [0.06-0.2]**	**-89**	**<0.001**
	**75-84**	**0.22 [0.17-0.28]**	**-78**	**<0.001**
	**≥85**	**0.24 [0.2-0.29]**	**-76**	**<0.001**

Table S10 Comparison of RSV-confirmed Hospitalization Incidence Rates Pre- and Post-COVID-19 across Countries and Age Groups including season 2019/20. This table presents the risk ratios (RR) comparing RSV-confirmed hospitalization incidence rates before (2016/17–2019/20) and after COVID-19 across different countries and age groups. It provides estimates of how hospitalization rates have changed post-COVID-19, along with confidence intervals (CI) and p-values to assess statistical significance.

- **RR (Risk Ratio):** A value above 1 indicates an increased incidence post-COVID-19, while a value below 1 suggests a decline.
- **post-COVID-19 Increase/Decrease (%):** Represents the percentage change, calculated as *1 – RR* (if RR > 1) or *RR – 1* (if RR < 1), then multiplied by 100. A negative value indicates a decline.
- **AvgIRPre:** The average (± standard deviation) of hospital admission rates pre-COVID-19.
- **AvgIRPost:** The average (± standard deviation) of hospital admission rates post-COVID-19.
- **Significant changes (p-value < 0.05):** Marked in blue to highlight statistically meaningful differences.

Country	Age group	RR	post-COVID-19 Increase/Decrease (%)	p-value
Denmark	18-64y	2.38 [0.57-9.97]	138	0.234
	65-74y	1.48 [0.79-2.8]	48	0.223
	75-84y	1.27 [0.83-1.95]	27	0.271
	85+y	1.34 [1-1.8]	34	0.053
Finland	18-64y	0.82 [0.41-1.65]	-18	0.581
	65-74y	0.78 [0.58-1.07]	-22	0.12
	**75-84y**	**0.49 [0.4-0.61]**	**-51**	**<0.001**
	**85+y**	**0.52 [0.45-0.6]**	**-48**	**<0.001**
Scotland	18-64y	0.5 [0.16-1.55]	-50	0.231
	**65-74y**	**0.5 [0.29-0.85]**	**-50**	**<0.001**
	**75-84y**	**0.66 [0.48-0.93]**	**-34**	**0.017**
	**85+y**	**0.56 [0.44-0.7]**	**-44**	**<0.001**
Spain-Valencia	**18-64y**	**0.31 [0.11-0.91]**	**-69**	**0.032**
	**65-74y**	**0.12 [0.07-0.23]**	**-88**	**<0.001**
	**75-84y**	**0.26 [0.21-0.33]**	**-74**	**<0.001**
	**85+y**	**0.28 [0.24-0.33]**	**-72**	**<0.001**

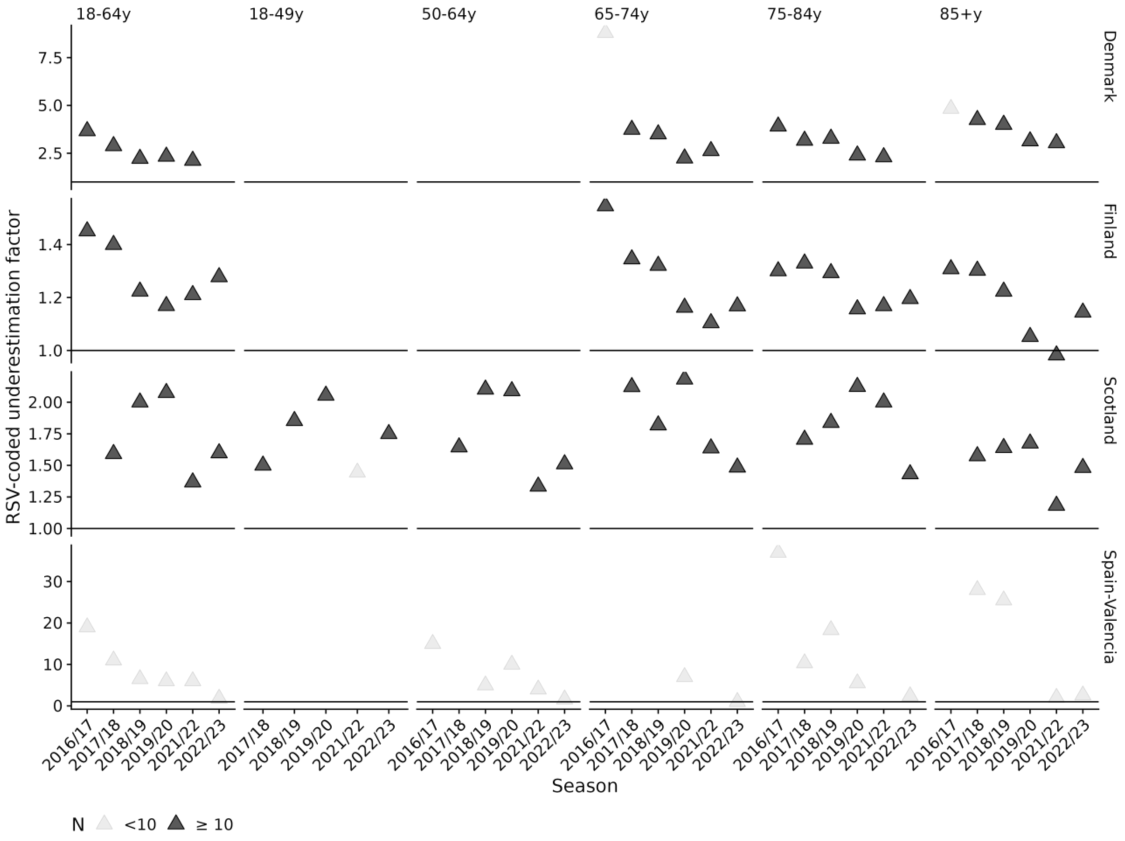

Figure S10 RSV-coding practices and its underestimation. The RSV-coded underestimation factor(y-axis) has been obtained by dividing in each season (x-axis), country (vertical subpanels) and age group (horizontal subpanels) the corresponding RSV-confirmed admission rate by the corresponding

RSV-coded admission rate. Numbers above the white line indicate that the incidence estimated with RSV-coded admissions is lower than the one estimated with RSV-confirmed for the corresponding country, age group and season. Groups with less than 10 patients in the RSV-coded dataset are shown in light grey.

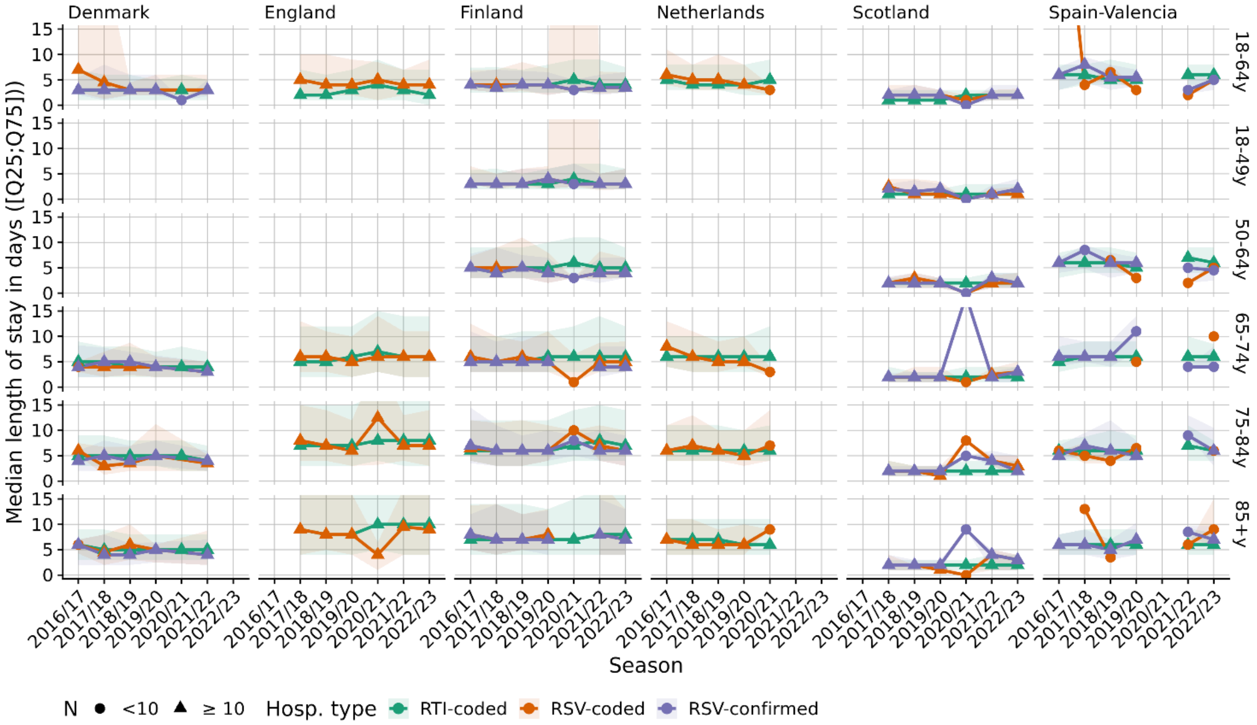

Figure S11 Hospitalisations median length of stay. Median LOS (y-axis) and 25/75 quartiles (shadow area) of all hospital admissions per country, age group and seasons (x-axis). Each hospitalisation type is shown with a different colour (green: RTI admissions, orange: RSV-coded and violet – RSV-confirmed admissions). If the estimate is based on less than 10 patients the shape is represented as a dot, whereas if the estimate is based on more than 10 patients the shape is represented as a triangle. Outliers (18-64 age group and RSV-coded in Spain-Valencia: 52 days [1 patient] and 65-74 age group and RSV-confirmed in Scotland [2 patients]: 18 days) are not shown to allow for readability of the graph.

Table S11 ICU admission rates. Average (±SD) RTI and RSV-associated ICU admissions in England of the seasons pre COVID-19 and of the seasons post COVID-19 stratified by age group and diagnosis group. The proportion of RTI/RSV-coded ICU admissions to the total number of RTI or RSV-coded admissions is also shown.

England			
**Age group**	**COVID-19**	**RTI**	**RSV-coded**
18-64	Pre	12,468 (12,176,12,760), 5.8%	65 (42,88), 7.2%
	Post	10,318 (4,931,15,705), 3.8%	47 (40,54), 3.0%
65-74	Pre	7,372 (7,040,7,704), 6.1%	46 (39,53), 7.9%
	Post	4,939 (2,664,7,214), 3.8%	22 (19,25), 2.1%
75-84	Pre	6,338 (6,145,6,531), 3.7%	27 (16,38), 3.9%
	Post	3,716 (2,336,5,096), 1.9%	12 (8,16), 0.9%
≥ 85	Pre	1,996 (1,899,2,093), 1.2%	5 (5,5), 0.9%
	Post	882 (628,1,136), 0.5%	5 (4,6), 0.4%
**Diagnosis groups**			
Bronchitis&Bronchiolitis	Pre	91 (89,93), 1%	4 (3,5), 4.4%
	Post	50 (44,56), 0.8%	2 (1,3), 1.2%
Unspecified LRTI	Pre	3,264 (3,143,3,385), 1.9%	10 (9,11), 1.7%
	Post	1,280 (974,1,586), 1.1%	8 (7,9), 0.8%
URTI	Pre	420 (414,426), 0.9%	2 (1,3), 1.6%
	Post	201 (173,229), 0.5%	1 (0,2), 0.7%
Pneumonia&Influenza	Pre	24,053 (23,948,24,158), 5.5%	97 (71,123), 8%
	Post	11,342 (9,096,13,588), 3.4%	40 (32,48), 2.0%
SARSCOV-2	Post	1,914 (1,354,2,474), 1.2%	1 (0,2), 1.7%
> 1 Diagnosis	Pre	321 (302,340), 3.8%	6 (4,8), 5.7%
	Post	5,043 (1,159,8,927), 4.8%	9 (9,9), 2.3%

Table S12 Case fatality rates. Average (±SD) RTI and RSV-associated admissions that end up in death in England, Finland and Spain-Valencia of the seasons pre COVID-19 and of the seasons post COVID-19 stratified by age group and diagnosis group. The case fatality rate to the total number of RTI or RSV-coded/confirmed admissions is also shown.

		England		Finland		
**Age group**	**COVID-19**	**Avg. RTI (±SD), CFR**	**Avg. RSV-coded (±SD), CFR**	**Avg. RTI (±SD), CFR**	**Avg. RSV-coded (±SD), CFR**	**Avg. RSV-confirmed (±SD), CFR**
18-64	Pre	8,391 (8,159,8,623), 3.9%	30 (29,31), 3.3%	481 (456,506), 3.7%	4 (1,7), 2.8%	5 (1,9), 2.5%
	Post	10,550 (9,182,11,918), 3.9%	54 (12,96), 3.5%	402 (175,629), 3.5%	1 (0,2), 0.6%	2 (2,2), 1%
65-74	Pre	12,660 (12,048,13,272), 10.4%	43 (40,46), 7.4%	995 (967,1023), 8.6%	7 (-1,15), 4%	8 (-2,18), 3.5%
	Post	14,282 (13,243,15,321), 10.9%	86 (15,157), 8.2%	958 (550,1366), 9.2%	4 (2,6), 2%	4 (0,8), 2.2%
75-84	Pre	24,388 (22,683,26,093), 14.1%	54 (40,68), 7.8%	1,910 (1,767,2,053), 12.2%	13 (-1,27), 5.7%	20 (-3,43), 6.5%
	Post	28,196 (28,170,28,222), 14.6%	152 (4,300), 10.8%	1,736 (1,049,2,423), 11.5%	7 (0,14), 3.5%	8 (0,16), 3.4%
≥ 85	Pre	34,054 (30,409,37,699), 19.8%	69 (49,89), 11.9%	2,559 (2,396,2,722), 17.8%	30 (3,57), 12.3%	38 (-4,80), 12.3%
	Post	35,145 (34,267,36,023), 19.7%	219 (-19,457), 17.6%	2,110 (1,313,2,907), 16.6%	12 (3,21), 6.2%	12 (1,23), 6.2%
**Additional age groups**						
18-49	Pre			69 (61,77), 1.2%	1 (0,2), 2.9%	1 (0,2), 1.9%
	Post			72 (44,100), 1.4%	0 (0,0), 0%	1 (0,2), 1.3%
50-64	Pre			412 (381,443), 5.5%	3 (1,5), 2.8%	4 (1,7), 2.83%
	Post			330 (131,529), 5.2%	1 (0,2), 1.0%	1 (0,2), 0.9%
**Diagnosis groups**						
Bronchitis&Bronchiolitis	Pre	111 (100,122), 1.2%	2 (1,3), 2.2%	84 (75,93), 2.5%	13 (3,23), 4%	11 (2,20), 3.2%
	Post	113 (107,119), 1.8%	6 (1,11), 3.6%	26 (8,44), 2.5%	4 (2,6), 1.6%	4 (2,6), 1.6%
Unspecified LRTI	Pre	7,393 (6,868,7,918), 4.2%	16 (14,18), 2.7%	51 (48,54), 4.6%	0 (0,0), 0%	0 (0,0), 0%
	Post	5,505 (5,371,5,639), 4.5%	35 (6,64), 3.4%	27 (14,40), 5.2%	0 (0,0), 0%	0 (0,0), 0%
URTI	Pre	148 (143,153), 0.3%	0 (-1,1), 0%	44 (35,53), 1.2%	0 (0,0), 0%	1 (0,2), 3.1%
	Post	124 (102,146), 0.3%	4 (0,8), 2.6%	18 (12,24), 1.2%	0 (0,0), 0%	1 (0,2), 5.6%
Pneumonia&Influenza	Pre	71,128 (67,023,75,233), 16.2%	134 (112,156), 11.1%	5,651 (5,369,5,933), 12.4%	32 (6,58), 9.0%	50 (0,100), 9.0%
	Post	55,010 (52,307,57,713), 16.5%	304 (86,522), 14.8%	2,836 (1,610,4,062), 11.7%	12 (5,19), 3.4%	14 (5,23), 3.2%
SARSCOV-2	Post	8,010 (7,914,8,106), 4.8%	5 (0,10), 8.3%	1,459 (1,300,1,618), 8.9%	0 (0,0), 0%	4 (2,6), 22.2%
> 1 Diagnosis	Pre	680 (620,740), 8.1%	12 (11,13), 11.4%	113 (96,130), 8.9%	8 (2,14), 7.2%	8 (2,14), 6.7%
	Post	19,330 (15,179,23,481), 18.2%	75 (13,137), 18.8%	842 (663,1,021), 14.1%	6 (2,10), 6.4%	7 (4,10), 7.1%

References

1. Lynge E, Sandegaard JL, Rebolj M. The Danish National Patient Register. Scand J Public Health. 2011;39(7 Suppl):30-3.
2. Pedersen CB. The Danish Civil Registration System. Scand J Public Health. 2011;39(7 Suppl):22-5.
3. Bliddal M, Broe A, Pottegård A, Olsen J, Langhoff-Roos J. The Danish Medical Birth Register. European Journal of Epidemiology. 2018;33(1):27-36.
4. Helweg-Larsen K. The Danish Register of Causes of Death. Scand J Public Health. 2011;39(7 Suppl):26-9.
5. Denmark S. Population of Denmark 2024 [04 march 2024]. Available from: https://www.statistikbanken.dk/statbank5a/default.asp?w=1920.
6. Hospital Episode Statistics. NHS Digital 2023 [04 March 2024]. Available from: https://digital.nhs.uk/data-and-information/data-tools-and-services/data-services/hospital-episode-statistics.
7. Office for National Statistics. 2021 census estimates classifying usual residents in England and Wales by single year of age London: United Kingdom Office for National Statistics; 2024 [July 2023]. Available from: https://www.ons.gov.uk/peoplepopulationandcommunity/populationandmigration/populationestimates/datasets/estimatesofthepopulationforenglandandwales.
8. Office for National Statistics. Mid-2001 to mid-2020 detailed population estimates for England and Wales London: United Kingdom Office for National Statistics; 2024 [July 2023]. Available from: https://www.ons.gov.uk/peoplepopulationandcommunity/populationandmigration/populationestimates/datasets/estimatesofthepopulationforenglandandwales.
9. eDRIS. Electronic Data Research and Innovation Service (eDRIS) 2023 [updated 18 December 202304 march 2024]. Available from: https://publichealthscotland.scot/services/data-research-and-innovation-services/electronic-data-research-and-innovation-service-edris/overview/what-is-edris/.
10. Gao C, McGilchrist M, Mumtaz S, Hall C, Anderson LA, Zurowski J, et al. A National Network of Safe Havens: Scottish Perspective. J Med Internet Res. 2022;24(3):e31684.
11. National Record of Scotland. Population, migration and households: National Records of Scotland; 2024 [10 September 2024]. Available from: https://www.nrscotland.gov.uk/statistics-and-data/population-migration-and-households/#).
12. Statistics Netherlands. Microdata: Conducting your own research 2024 [04 March 2024]. Available from: https://www.cbs.nl/en-gb/our-services/customised-services-microdata/microdata-conducting-your-own-research.
13. Mira-Iglesias A, López-Labrador FX, Baselga-Moreno V, Tortajada-Girbés M, Mollar-Maseres J, Carballido-Fernández M, et al. Influenza vaccine effectiveness against laboratory-confirmed influenza in hospitalised adults aged 60 years or older, Valencia Region, Spain, 2017/18 influenza season. Eurosurveillance. 2019;24(31).
14. Mira-Iglesias A, López-Labrador FX, Guglieri-López B, Tortajada-Girbés M, Baselga-Moreno V, Cano L, et al. Influenza vaccine effectiveness in preventing hospitalisation of individuals 60 years of age and over with laboratory-confirmed influenza, Valencia Region, Spain, influenza season 2016/17. Eurosurveillance. 2018;23(8).
15. Mira-Iglesias A, Demont C, López-Labrador FX, Mengual-Chuliá B, García-Rubio J, Carballido-Fernández M, et al. Role of age and birth month in infants hospitalized with RSV-confirmed disease in the Valencia Region, Spain. Influenza and Other Respiratory Viruses. 2022;16(2):328-39.
16. Puig-Barberà J, García-De-Lomas J, Díez-Domingo J, Arnedo-Pena A, Ruiz-García M, Limón-Ramírez R, et al. Influenza Vaccine Effectiveness in Preventing Influenza A(H3N2)-Related Hospitalizations in Adults Targeted for Vaccination by Type of Vaccine: A Hospital-

Based Test-Negative Study, 2011–2012 A(H3N2) Predominant Influenza Season, Valencia, Spain. PLoS ONE. 2014;9(11):e112294.
17. Commission Implementing Decision (EU) 2018/ 945. Decisions. Official Journal of the European Union. 2018:L170/1 - L/74.
18. Generalitat Valenciana Department of Health. Population Information System (SIP) 2025 [10 February 2025]. Available from: https://www.san.gva.es/es/web/tarjeta-sanitaria/sistema-de-informacion-poblacional-sip.
